# A poxvirus pseudokinase represses viral DNA replication via a pathway antagonized by its paralog kinase

**DOI:** 10.1371/journal.ppat.1007608

**Published:** 2019-02-15

**Authors:** Annabel T. Olson, Zhigang Wang, Amber B. Rico, Matthew S. Wiebe

**Affiliations:** 1 Nebraska Center for Virology, University of Nebraska, Lincoln, NE, United States of America; 2 School of Biological Sciences, University of Nebraska, Lincoln, NE, United States of America; 3 School of Veterinary Medicine and Biomedical Sciences, University of Nebraska, Lincoln, NE, United States of America; University of Utah, UNITED STATES

## Abstract

Poxviruses employ sophisticated, but incompletely understood, signaling pathways that engage cellular defense mechanisms and simultaneously ensure viral factors are modulated properly. For example, the vaccinia B1 protein kinase plays a vital role in inactivating the cellular antiviral factor BAF, and likely orchestrates other pathways as well. In this study, we utilized experimental evolution of a B1 deletion virus to perform an unbiased search for suppressor mutations and identify novel pathways involving B1. After several passages of the ΔB1 virus we observed a robust increase in viral titer of the adapted virus. Interestingly, our characterization of the adapted viruses reveals that mutations correlating with a loss of function of the vaccinia B12 pseudokinase provide a striking fitness enhancement to this virus. In support of predictions that reductive evolution is a driver of poxvirus adaptation, this is clear experimental evidence that gene loss can be of significant benefit. Next, we present multiple lines of evidence demonstrating that expression of full length B12 leads to a fitness reduction in viruses with a defect in B1, but has no apparent impact on wild-type virus or other mutant poxviruses. From these data we infer that B12 possesses a potent inhibitory activity that can be masked by the presence of the B1 kinase. Further investigation of B12 attributes revealed that it primarily localizes to the nucleus, a characteristic only rarely found among poxviral proteins. Surprisingly, BAF phosphorylation is reduced under conditions in which B12 is present in infected cells without B1, indicating that B12 may function in part by enhancing antiviral activity of BAF. Together, our studies of B1 and B12 present novel evidence that a paralogous kinase-pseudokinase pair can exhibit a unique epistatic relationship in a virus, perhaps serving to enhance B1 conservation during poxvirus evolution and to orchestrate yet-to-be-discovered nuclear events during infection.

## Author summary

Vaccinia virus is the archetype member of the Poxviridae family. This virus encodes ~200 genes that contribute to viral propagation in the cytoplasm and coordinate restriction of the host antiviral response through mechanisms that are incompletely understood. Notably, the B1 kinase inhibits a highly conserved antiviral protein, allowing viral DNA replication to proceed in the cytoplasm. To better understand B1 driven signaling, we used experimental evolution of a B1 deletion virus to identify viral genes within shared signaling pathways. Herein we describe our success in isolating adapted viruses and the discovery of a genetic link between B1 and the related B12 gene. Thus this study illustrates the utility of experimental evolution to discover novel digenic relationships. Interestingly, we also elucidated an unexpected regulatory intersection between the B1 kinase and B12 pseudokinase that likely masked discovery of the repressive function of B12 during previous studies of this factor. As kinase-pseudokinase protein families are abundant in many forms of life, the genetic and functional relationship between B1 and B12 that we uncovered is likely of broad biological relevance. Further investigation of individual genes and gene families in multigenic knockout backgrounds may uncover similar masked phenotypes in a broad range of model systems.

## Introduction

Protein kinases regulate the function of a large fraction of cellular proteins, governing numerous molecular processes [1–3]. However, much remains unknown about how this class of proteins is regulated and what evolutionary mechanisms may have driven their conservation in all kingdoms of life as well as viruses. Investigation of kinases encoded by poxviruses has provided fascinating insights into how these factors dysregulate host signaling pathways and orchestrate viral protein function. Expressed early during infection, the product of the vaccinia *B1R* gene encodes the B1 Ser/Thr kinase vital for productive infection with a clear role in impairing at least one facet of the host antiviral response [4–6]. B1 homologs are highly conserved within the members of the Poxviridae family that infect mammals, with the only exceptions being the Molluscipoxvirus and Parapoxvirus genera. Interestingly, a group of eukaryotic kinases have homology (~40% amino acid identity) to the vaccinia B1 protein [7–10]. These proteins are named vaccinia related kinases (VRKs) and have been found to share at least one common substrate with B1, demonstrating that the B1/VRK enzymes represent an intersection of viral and host signaling pathways. Much of what we know regarding the function of B1 is based on studies of temperature-sensitive mutant viruses with point mutations in the B1 locus [4, 5, 11–17] as well as a recently described B1-knockout virus [18]. Phenotypically, progeny of these B1-deficient viruses are markedly reduced in number during infection, due to profound defects in viral DNA replication [4, 5, 15, 18]

To ensure replication of the vaccinia genome, it is critical that B1 phosphorylate the cellular protein BAF, encoded by the *BANF1* gene. BAF is a highly conserved DNA-binding protein with essential cellular functions related to maintaining genomic integrity via diverse pathways [19]. For example, BAF is capable of intercepting cytoplasmic DNA and assembling higher-order DNA-protein assemblies [20, 21]. This allows BAF to strongly inhibit vaccinia virus DNA replication [6] and intermediate transcription [12]. However, the host defense activity of BAF against vaccinia virus is dependent on its DNA-binding property, which can be blocked through phosphorylation mediated by B1 [6, 22], thus allowing poxvirus DNA replication to proceed. Although BAF phosphorylation by B1 clearly enhances viral fitness, genetic and biochemical studies indicate that B1 likely contributes to poxviral replication via other pathways as well. For example, it has been discovered that RACK1 (receptor for activated C kinase) is phosphorylated in a B1 dependent manner, triggering a selective advantage for translation of viral RNAs that is postulated to enhance viral fitness late in infection [23]. Some other known substrates of the B1 kinase include the ribosomal Sa and S2 proteins [24] as well as the viral H5 proteins [14, 25], each of which can be directly phosphorylated by B1 in vitro and is modified in a B1-dependent manner in infected cells. However, although it has been known for some time that these proteins are substrates of B1, whether their phosphorylation by B1 is beneficial during the poxvirus lifecycle remains unclear.

In this study we utilized experimental evolution of a B1 deletion virus to search for novel pathways through which B1 functions. This approach leverages the natural errors that occur during vaccinia DNA replication to introduce variants that can suppress the fitness defect caused by a deleted gene. Vaccinia mutants with enhanced replicative capacity become enriched in the population during serial passage and can then be identified using whole genome sequencing. In previous studies of other viral proteins, experimental evolution has yielded strong evidence of genetic interaction between viral gene products as well as fascinating insights into novel mechanisms of poxviral evolution. For example, suppression of growth defective phenotypes in other vaccinia mutants occurred via rapid introduction of multiple copies of an existing poxviral gene, resulting in a gain of function by those viruses [26, 27]. Other gain of function mutations that have been observed include single nucleotide changes in coding regions that introduce single amino acid changes [28, 29]. In contrast, our experiments here with the B1 deletion virus reveal that a mutation resulting in loss of function provides a striking fitness enhancement to this virus. Validating predictions that gene loss can be a driver of poxvirus evolution [30], this is clear experimental evidence that such loss can be beneficial in certain contexts.

The loss of function mutation that suppressed the B1 deletion phenotype was mapped to the B12 locus of the vaccinia genome, where indels introduced frameshifts into the coding region of that gene. B12 is 36% identical to B1 at the amino acid level and is thus a paralog of that kinase likely to have resulted from an earlier gene duplication event of a B1-like ancestor [17, 31]. B1 is likely to have been the earlier of the two genes expressed, as B1 orthologs are found in most of the Chordopoxviranae subfamily, while B12 is restricted to members of the Orthopoxvirus genus and is not present in any viruses lacking B1. A hallmark of all poxvirus B12 orthologs, including that of vaccinia, is that they possess amino acid variants at key residues predicted to be critical for catalytic activity [17] and therefore fits the definition given by Manning and colleagues for pseudokinases [1]. Pseudokinases are a subgroup of protein kinases found to play myriad regulatory roles in signaling in metazoans, but in many cases do not possess enzymatic activity themselves [32, 33]. Here, we present multiple lines of evidence demonstrating that expression of wild-type B12 leads to a striking reduction in fitness of viruses with a defect in B1. Importantly, while mutation or depletion of B12 can rescue the B1 defect in viral DNA replication in multiple cell types, altering the levels of B12 has no apparent impact on wild-type virus or other mutant viruses. From these data we infer that the inhibitory mechanism executed by B12 is repressed by the B1 kinase. This signaling relationship bears similarity to that found in gene pairs such as toxin-antitoxin genes in bacteria [34–37] or in poison-antidote genetic elements in higher organisms which augment the fitness value of the antidote gene to an organism [38–40]. Additional investigation in search of a mechanism of action for B12 revealed that it primarily localizes to the nucleus, a property only rarely found among poxviral proteins. Furthermore, the adapted virus containing a B12 mutation exhibits reduced sensitivity to BAF overexpression, suggesting that B12 may function, at least partly, via a BAF dependent mechanism. Together, our studies of B1 and B12 present novel evidence that a paralogous kinase-pseudokinase pair can exhibit this type of epistatic relationship in a virus, perhaps serving to enhance B1 conservation during poxvirus evolution and to orchestrate yet-to-be-discovered nuclear events during infection.

## Results

### Fitness gains observed following adaption of the ΔB1 virus correlate with an indel mutation within the B12R gene

The vaccinia virus B1 kinase is a critical positive regulator of vaccinia DNA replication. Specifically, B1 inhibits the BAF antiviral factor, which can otherwise restrict viral DNA replication and subsequent gene expression [6]; however, much remains unknown about how B1 may regulate other viral factors during infection. In a recent study of B1 function, our laboratory generated a mutant vaccinia virus (ΔB1 virus) in which the *B1R* gene was deleted by homologous recombination [18]. As expected, without this viral kinase, growth of the ΔB1 virus was severely impaired compared to WT virus. Intriguingly, the fact that some progeny virus could be isolated following infection with the ΔB1 virus suggested to us that it may be amenable for use in a screen for second site suppressors using an experimental evolution protocol. If successful, this approach could reveal novel genetic linkages between *B1R* and other viral genes.

**Fig 1:**
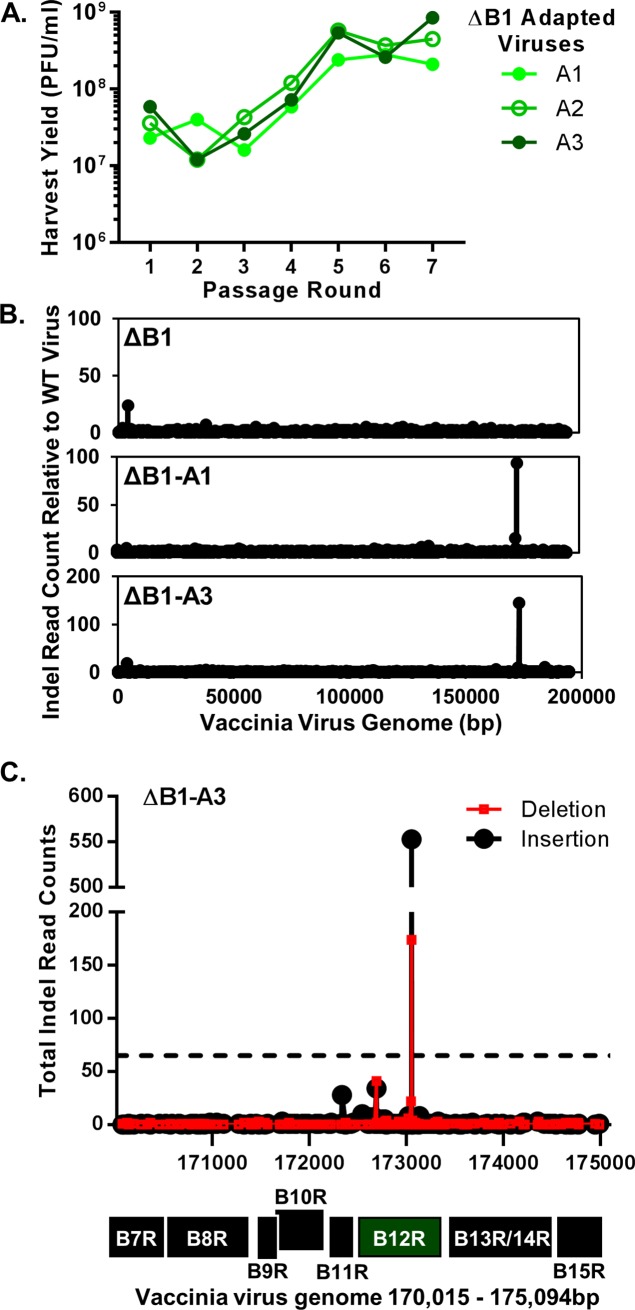
Adaptation of ΔB1 virus and identification of mutation within the B12R gene. (A) ΔB1 virus was serially passaged in CV1 cells in triplicate and named A1-A3 for adapted ΔB1 viruses. Virus harvested at each passage was titrated on CV1-B1myc cells. (B) Deep sequencing data for WT (Wiebe), ΔB1, ΔB1-A1, and ΔB1-A3 viruses was used to graph insertion/deletion mutations at each nucleotide site for the entire vaccinia genome when comparing ΔB1, ΔB1-A1, and ΔB1-A3 viruses to the change in indel mutations of the WT (Wiebe) compared to the WT WR (reference sequence). (C) Graphed insertion/deletion mutations for ΔB1-A3 compared to WT WR (reference sequence) for reads 170,015–175,094bp. The dotted line indicates indel mutations that occur in 5% of the total reads at a single nucleotide. Indel mutations above 5% were considered significant mutations in the mixed ΔB1 adapted virus population. Locations of encoded genes are labeled below, corresponding to the base pairs on the x-axis of the graph.

The experimental evolution of ΔB1 was conducted by iterative passage of the virus at a low multiplicity of infection (MOI) of 0.1 on non-complementing CV1 cells. Each infection was allowed to proceed for two days prior to harvest. After each passage the viral yield was determined by plaque assay titration on complementing, B1 expressing CV1 (CV1-B1myc) cells characterized previously [18]. Virus titrations on the CV1-B1myc cells allowed for accurate quantification of serially passaged ΔB1 virus, while no visible plaques could form on the non-complementing CV1 cells. The process of harvesting, titration on CV1-B1myc cells, and reinfection of CV1 cells at low MOI was carried out for seven passage rounds. Virus yield at each passage was graphed for the A1, A2, and A3 lineages of adapted ΔB1 virus (Fig 1A). During these serial passages, the yield of the ΔB1 virus showed a notable 10-fold increase in titer between passage rounds 3 and 5, suggesting the emergence of a rescued virus in all three, independent replicates. Using quantitative PCR we verified that the *B1R* gene remained undetected in our serially passaged viruses (S1A Fig), thus confirming that reintroduction of B1 is not responsible for the rescue of the passaged ΔB1 virus.

Next, we predicted that the 10-fold increase in viral yield may be sufficient to permit spread through non-complementing cells and allow plaque formation. To test this prediction, we infected CV1 control cells and CV1-B1myc cells with 200 plaque forming units (PFU) per well of WT, ΔB1, and each passage of adapted ΔB1 virus from lineage A1, then fixed and stained the cells at 72h post infection. Starting at passage three, the adapted cultures contained virus that formed plaques which were smaller than WT, but clearly visible on non-complementing CV1 cells (S1B Fig, top row). As expected, adapted ΔB1 virus plaque size was increased in cells expressing B1 in trans (S1B Fig, bottom row). These data confirm the rescue of the adapted ΔB1 virus in non-complementing CV1 cells, although the smaller plaque phenotype of the adapted ΔB1 virus suggests that it is less fit than the WT virus in this assay.

With three independently adapted ΔB1 viruses in hand, we sought to identify significant genetic alterations within the adapted viruses as compared to the ΔB1 virus. To this end, DNA isolated from the WT (Wiebe laboratory), ΔB1, and adapted ΔB1 viruses A1 and A3 (passage round 7) were subjected to 150 base pair paired end sequencing using a MiSeq V2 instrument. Following guided assembly, WT (Wiebe), ΔB1, ΔB1-A1, and ΔB1-A3 sequences were compared to the WT Western Reserve reference genome (NC_006998.1) in the NCBI database. One previously characterized mechanism of virus adaptation involves the development of genomic ‘accordions’ in which gene amplifications occur for a specific locus, permitting a dose dependent compensation by a complementary gene [26, 27]. However, the ΔB1-A1 and ΔB1-A3 virus sequence data lacked evidence of genomic accordions, which would have been identified by large increases of sequence read counts for a particular region in the adapted genomes compared to WT and ΔB1 controls. Instead we observed a consistent read count depth across the entire genome for both adapted and control viruses. We also analyzed the specific read calls at each nucleotide position in the complete genome for evidence of single nucleotide polymorphisms (SNPs). For this analysis we made sequence comparisons between WT (Wiebe) to WT WR (reference sequence), ΔB1 to WT (Wiebe), and ΔB1mutB12 to ΔB1. From the genome analyses of ΔB1-A1 and ΔB1-A3 viruses, only one non-synonymous mutation was identified, which was found solely in the ΔB1-A1 virus. This mutation identified in ΔB1-A1 is a G to A substitution expected to cause a Gly215 to Asp mutation within the *B12R* coding region. Based on the read depth for this specific nucleotide location, we determined that this point mutation is present in 18% of the reads from the ΔB1-A1 virus (S1C Fig and S1 Table). Finally, we quantified reads containing insertion/deletion (indel) mutations across the entire genome for ΔB1, ΔB1-A1, and ΔB1-A3 as compared to the changes in WT (Wiebe) from the WT WR (reference sequence) genome. To present the locations of indels graphically, comparisons of indel read counts are plotted along the y-axis for ΔB1 to WT (Fig 1B, top), ΔB1-A1 to WT (middle), and ΔB1-A3 to WT (bottom) and the nucleotide numbers of the reference genome along the x-axis. Strikingly, indel mutations were identified at identical nucleotide positions in the ΔB1-A1 and ΔB1-A3 comparisons to WT (Fig 1B, middle and bottom graph), but not for ΔB1 sequence comparison to WT (Fig 1B, top graph). Narrowing the x-axis to focus on the region of interest, we graphed the total number of indel mutations found between 170,015 and 175,094 base pairs for the ΔB1-A3 alignment to the ΔB1 genome (Fig 1C). The genes labeled below the x-axis indicate the genes encoded at the specific base pair regions. This graph depicts that the ΔB1-A3 virus population contains a significant spike in insertion and deletion mutations within the *B12R* gene. This spike of indel mutations corresponds to adenine 689 in the *B12R* gene, which is the start of an eight adenine sequence (S1 Table). This single site of indel mutations is present in approximately 48% and 65% of the reads for the ΔB1-A1 and ΔB1-A3 virus, respectively (S1 Table). Interestingly, either an insertion or deletion of an adenine at this site alters the predicted reading frame of the gene and thereby introduces a premature stop codon, resulting in a truncated B12 protein. In addition to the Illumina sequencing of mixed viral populations, targeted Sanger sequencing of the B12 locus was performed on 10 isolated plaques chosen from the three adapted cultures after two rounds of plaque purification. As a result, we found that all ten isolated plaques contain either an indel mutation at B12 nucleotide 689 or an indel at another earlier position in *B12R* (S1 Table). Together these results highlight the significant frequency of mutations within the *B12R* gene, leading it to become our top candidate for a ΔB1 second site suppressor mutation.

The B12 protein is 283 amino acids in length and shares 36% amino acid identity to the vaccinia B1 kinase [17, 31]. Akin to the B1 kinase, the B12 protein is homologous to the cellular vaccinia related kinases (VRKs). Furthermore, the viral *B12R* gene is expressed early in infection, like the B1 kinase, but differs from the B1 kinase in that it lacks catalytic activity [17, 41]. Proteins that possess sequence and structural similarity to active kinases, but lack phosphotransferase activity due to alterations in key catalytic residues are abundant in all forms of life and are commonly referred to as pseudokinases. Although B12 is a pseudokinase and B1 paralog, the function of B12 during infection remains an enigma to date, as previous studies revealed no phenotypic defect for a mutant vaccinia virus missing 83% of the *B12* gene [41, 42].

### The ΔB1mutB12 virus exhibits rescued DNA replication and viral yield in multiple cell lines

**Fig 2:**
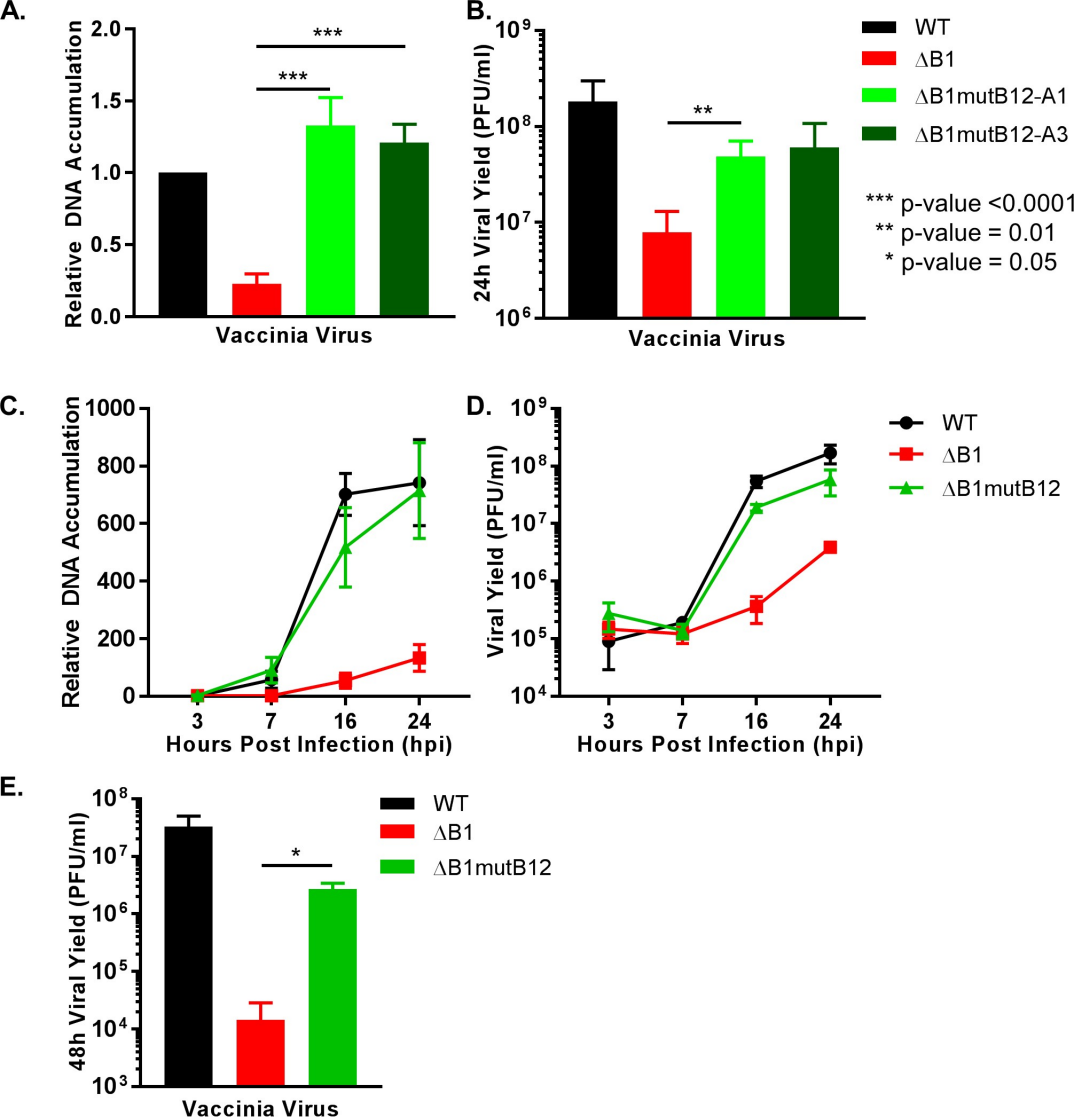
Rescued DNA replication block and viral yield for ΔB1mutB12 virus in CV1 cells. (A) CV1 cells infected with WT (black), ΔB1 (red), ΔB1mutB12-A1 (light green), ΔB1mutB12-A3 (dark green) at a MOI of 3 were harvested 24h post infection for qPCR of relative DNA accumulation or (B) for titration on CV1-B1myc cells for viral yield. (C) WT (black), ΔB1 (red), or ΔB1mutB12-A3 (green) infections of CV1 cells were performed at a MOI of 3 and harvested at 3, 7, 16, or 24h post infection for relative DNA accumulation or (D) viral yield quantification on CV1-B1myc cells. (E) A multi-step viral yield assay was completed by infecting CV1 cells at a MOI of 0.01 with WT (black), ΔB1 (red) or ΔB1mutB12-A3 (green) and harvested at 48h post infection for titration on CV1-B1myc cells.

As described above, the adapted ΔB1 virus (hereafter referred to as the ΔB1mutB12 virus) exhibited a visible plaque phenotype not observed for the ΔB1 virus. To investigate the extent of the ΔB1mutB12 rescued phenotype, we measured both DNA accumulation and viral yield during a single round of vaccinia virus replication in noncomplementing cells. Following synchronous infections at a MOI of 3, viral genome replication was measured by qPCR. Compared to the ΔB1 virus, the ΔB1mutB12-A1 (light green bars) and the ΔB1mutB12-A3 (dark green bars) viruses exhibit increased DNA accumulation at 24h, exceeding ΔB1 (red bars) levels by >5-fold in monkey CV1 cells (Fig 2A) and >18-fold in human HeLa cells (S2A Fig). Increases were also observed in human A549 cells (S2B Fig) and mouse L929 cells (S2C Fig), although to a lesser degree.

Regarding viral progeny, the ΔB1mutB12-A1 (light green bars) and ΔB1mutB12-A3 (dark green bars) exhibit rescued viral yield phenotypes in multiple cell lines when compared to the ΔB1 (red bars) virus. Specifically, the viral yields for ΔB1mutB12-A1 and -A3 viruses compared to ΔB1 levels were increased >6-fold in monkey CV1 cells (Fig 2B), >50-fold in human HeLa cells (S2D Fig), >6-fold in human A549 cells (S2E Fig), and >10-fold in mouse L929 cells (S2F Fig). Notably, despite a >50-fold rescue over the ΔB1 viral yield, the ΔB1mutB12-A1 and -A3 viruses remained at 11-fold (p-value = 0.0056) and 13-fold (p-value = 0.0051) lower levels than WT yields in HeLa cells (S2D Fig). Similarly, the ΔB1mutB12-A1 virus was 28-fold (p-value = 0.0474) lower than WT viral yield in L929 cells (S2F Fig), despite showing a >10-fold increase from ΔB1 levels. These results demonstrate a rescued viral yield phenotype for ΔB1mutB12-A1 and -A3 over ΔB1 levels in all cell lines, while remaining attenuated compared to WT virus.

To examine ΔB1mutB12-A3 virus yield at earlier time points, CV1 cells infected with WT (black line), ΔB1 (red line) or ΔB1mutB12-A3 (green line) virus were harvested early during infection to monitor input DNA (3hpi) and initial DNA replication (7hpi), while later time points were selected for the completion of DNA replication (16hpi) and the completion of vaccinia virus replicative cycle (24hpi). DNA accumulation at each time point demonstrated that the ΔB1mutB12-A3 virus replicated its genome at a rate similar to WT (Fig 2C). Next, virus samples were titrated on CV1-B1myc complementing cells to quantify viral yield at each time point. At early time points the ΔB1mutB12 viral yield levels were identical to WT levels. Similar to the 24h only data (Fig 2B), the ΔB1mutB12-A3 virus exhibited an almost 3-fold reduction in viral yield as compared to WT virus at late time points, although these differences were not statistically different (Fig 2D, 16 and 24hpi). Therefore, the WT virus and ΔB1mutB12 virus have only modest growth difference with respects to DNA accumulation and viral yield output in CV1 cells under these conditions.

The similar growth profiles of WT and ΔB1mutB12 viruses in CV1 cells at a MOI of 3 led us to question whether the same was true at lower concentrations of virus. For this next assay, cells were infected with a low MOI of 0.01 and allowed to propagate, spreading cell-to-cell, for 48h before harvest and titration on CV1-B1myc cells. From these infections, ΔB1mutB12 (green bar) viral yield was >180-fold higher than ΔB1 (red bar) virus (Fig 2E). Interestingly, the ΔB1mutB12-A3 (green bars) virus was attenuated 12-fold (p-value = 0.09) as compared to the WT (black bars) virus viral yield (Fig 2E). In summary, the ΔB1mutB12 virus exhibits a rescued DNA accumulation phenotype compared to ΔB1 in multiple species cell lines. The viral yield of the ΔB1mutB12 virus also indicates a recovered growth phenotype as compared to ΔB1 levels for all cell lines tested. Yet, the extent of ΔB1mutB12 attenuation compared to WT viral yield differed depending on the cell line and amount of virus used for infection.

### The B12ΔA690 protein is truncated and accumulates to lower levels than the wild-type B12 protein

**Fig 3:**
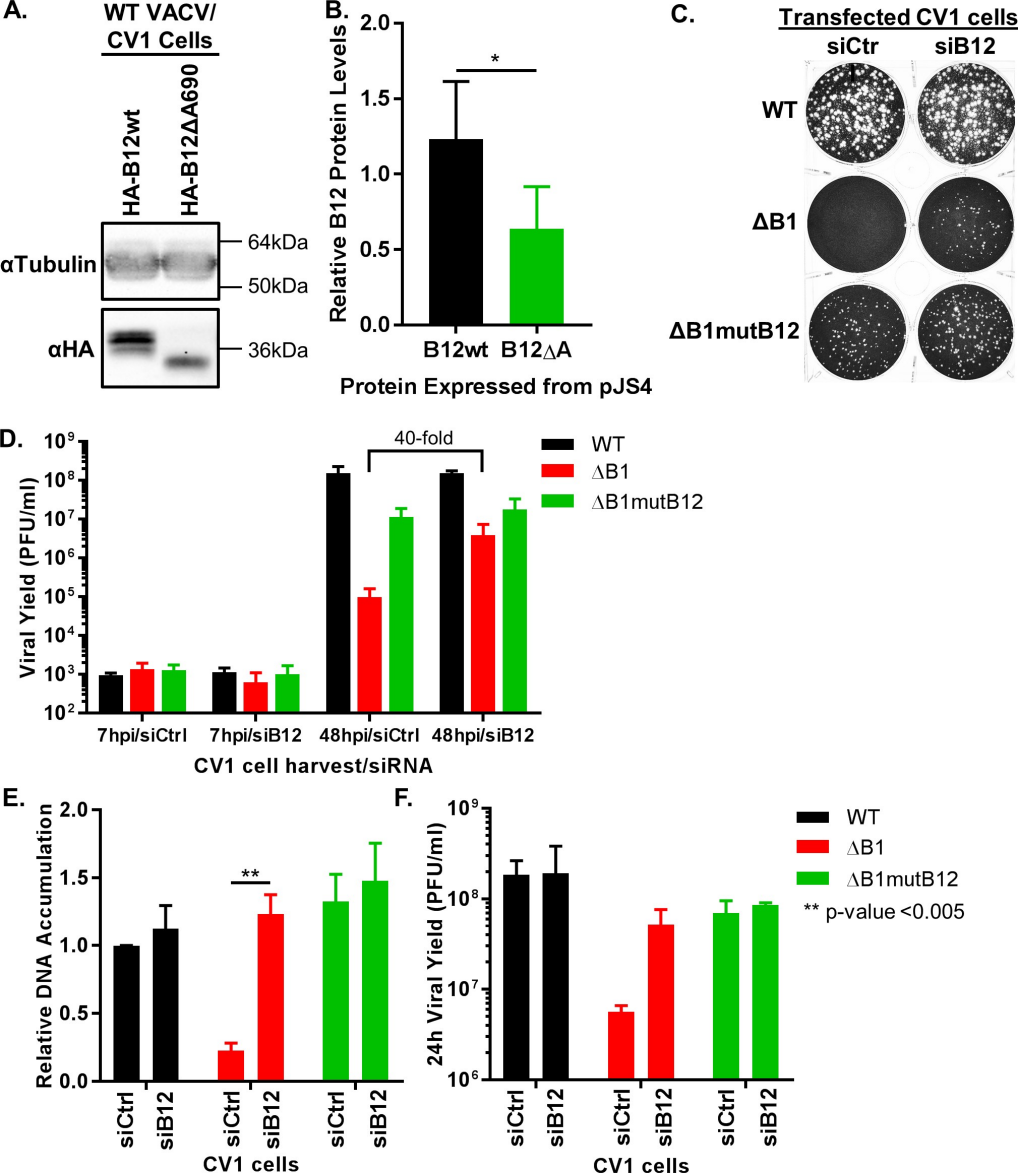
Depletion of B12 rescues ΔB1 virus growth in CV1 cells. (A) CV1 cells were transfected with pJS4-HA-B12wt or pJS4-HA-B12ΔA690 plasmid and infected 6h post transfection with WT virus at a MOI of 3. 24h post infection cells were harvested for immunoblot analysis. The HA-B12Δ690 represents the indel mutation within the ΔB1mutB12 virus *B12R* gene. (B) B12 proteins were expressed from the pJS4 vector during WT infection (representative immunoblot in Fig 1A). Relative protein levels for HA-B12wt and HA-B12ΔA690 were averaged from five independent experiments, and normalized to control protein levels set to 1. The denoted * p-value equals 0.02. (C) 200PFU/well WT, ΔB1, or ΔB1mutB12-A3 infections were carried out on CV1 cells 24h following transfection with siCtrl or siB12. Cells were fixed 72h post infection. (D) Multi-step viral yield assay was conducted in siCtrl or siB12 CV1 cells for WT (black), ΔB1 (red), and ΔB1mutB12-A3 (green) infections at a MOI of 0.01. Cells were harvested at 7h or 48h post infection and titration on CV1-B1myc cells. (E) Growth assays on siCtrl or siB12 transfected CV1 cells were completed for WT (black), ΔB1 (red), and ΔB1mutB12 (green) viruses at a MOI of 3 for relative DNA accumulation and (F) viral yield titration on CV1-B1myc cells.

The sequencing data for the ΔB1mutB12 viruses revealed a prevalent indel mutation within the 3’ end of the *B12R* gene that leads to a frame shift. This frameshift introduces a premature stop codon into the mRNA, which is predicted to translate into a protein missing about 45 amino acids from the C-terminus, about 16% of the total polypeptide. Studies on VRK1, a cellular homolog of B12 determined that removal of 18% of the protein from the C-terminus produces a protein that cannot be purified from *E*. *coli* [43] suggesting that this region is necessary for protein folding and/or stability. Based on this precedent and B12 sequence analysis, we hypothesized that the indel mutation within the *B12R* gene will lead to a truncated protein with reduced protein accumulation. To test this hypothesis, we PCR amplified the wild-type *B12R* vaccinia gene from the WT vaccinia virus and generated a *B12R* mutant in which a single adenine was deleted at nucleotide 690, corresponding to the site of the indel in ΔB1mutB12. Next, we cloned the vaccinia virus *B12R* gene or the *B12R* indel mutant with a HA epitope tag sequence at the 5’ end of the gene into the pJS4 vaccinia expression vector. CV1 cells were transfected with pJS4-HA-B12wt or pJS4-HA-B12ΔA690 plasmid DNA, synchronously infected with WT virus at a MOI of 3 and harvested 24h post infection to allow for saturation of late gene expression. Following immunoblot analysis, the representative immunoblot and cumulative bar graph show HA-B12ΔA690 protein levels were reduced as compared to HA-B12wt to 52% of the wild-type protein abundance (Fig 3A and 3B). The HA-B12ΔA690 band has a smaller molecular weight as shown by a faster migrating band than the HA-B12wt band (Fig 3A). Together, these results support the hypothesis that deletion of the adenine at nucleotide position 690 within the *B12R* gene results in a truncated protein, which is expressed at reduced levels when compared to the wild-type B12 protein.

### Loss of B12 through depletion rescues the ΔB1 and ts2 growth phenotype, but not other viruses with restricted DNA replication phenotypes

The results thus far confirm that HA-B12ΔA690 is both truncated and may be less stable than the HA-B12wt protein. We posited two possible scenarios to explain how the enhanced replication of ΔB1mut12 may be mediated by the truncation and reduced abundance of B12. Either a proviral activity of B12 is increased in ΔB1mutB12 due to the absence of a regulatory domain lost after truncation, or wild-type B12 is capable of a repressive activity that is no longer present in the ΔB1mutB12 virus. To distinguish between these gain of function versus loss of function scenarios for the *B12R* indel mutation, we decided to test how ΔB1 growth was impacted by B12 depletion mediated by siRNA targeting of B12 mRNA. First, as controls, B12 mRNA levels for WT (black bars), ΔB1 (red bars), and ΔB1mutB12 (green bars) were quantified at 4h post infection in CV1 cells. Purified RNA was reverse transcribed to cDNA for qPCR quantitation of relative B12 mRNA levels using a specific primer and probe set (S3A Fig). Each virus expressed similar levels of relative B12 mRNA (S3B Fig, siCtrl). Second, we determined the level of B12 depletion using siRNA targeting B12 mRNA. Transfection of CV1 cells with siB12 prior to infection reduced relative B12 mRNA to <28% for each virus as compared to control cells (S3B Fig). We also verified that downstream *B13R* gene expression was not altered for the ΔB1mutB12 virus or during B12 mRNA depletion (S3C and S3D Fig) using two different B13 primer/probe sets (S3A Fig). We further validated that these primer probe sets were specific for B13 by demonstrating that they were sensitive to siRNAs specific to B13 (S3C and S3D Fig).

Upon successful B12 depletion using siRNA, we addressed the question of whether B12 loss of function rescues the ΔB1 growth phenotype. To test the rescue of ΔB1 plaque formation by depletion of B12, a plaque assay was carried out on CV1 siCtrl and siB12 treated cells during WT, ΔB1, and ΔB1mutB12 virus infections at 200 PFU/well (Figs 3C and S3E). We observed that WT and ΔB1mutB12 viruses can form plaques on CV1 siCtrl cells and similarly on CV1 siB12 cells. Strikingly, the ΔB1 virus that is unable to form plaques on CV1 siCtrl cells was able to form plaques in CV1 siB12 cells that were of a similar size as those present in the wells infected with ΔB1mutB12. To quantify this rescue in viral yield, CV1 cells were infected with WT, ΔB1, or ΔB1mutB12 virus at a low MOI of 0.01 for a multi-step growth assay. Infected cells were harvested at 7h and 48h post infection. At 7h post infection, each virus shows similar amounts of viral yield in CV1 siCtrl-treated cells and CV1 siB12-treated cells (Fig 3D). This measurement at 7h post infection is indicative of input virus. At 48h post infection, WT (black bars) and ΔB1mutB12 (green bars) yields remain constant between control and B12 depletion (Fig 3D). By comparison, the ΔB1 (red bar) virus increases 40-fold in the CV1 siB12 cells as compared to CV1 siCtrl cells (Fig 3D).

Next, we quantified the rescue of DNA accumulation and viral yield following B12 depletion during ΔB1 infection using a one-step viral growth assay. Both WT and ΔB1mutB12 DNA accumulation levels are not significantly increased during infection of CV1 siB12 cells as compared to CV1 siCtrl cells (Fig 3E). However, DNA accumulation of ΔB1 increases 5.4-fold in CV1 siB12 cells compared to CV1 siCtrl cells. Similarly, the viral yield for WT and ΔB1mutB12 viruses remains constant between CV1 siCtrl and siB12 infected cells while ΔB1 yields an increase of about 9-fold in CV1 siB12 cells as compared to siCtrl treated cells (Fig 3F). In summary, the replication assays of ΔB1 virus during B12 depletion indicate that the loss of B12 function rescues the ΔB1 phenotype. This siRNA study also refutes the gain of function scenario outlined earlier. Specifically, if the indel mutation within *B12R* resulted in a gain of function, then the depletion of the B12 mutant during ΔB1mutB12 infections should have restored the attenuated ΔB1 phenotype during these infections, but it did not (Fig 3E and 3F, compare siB12 ΔB1mutB12 to siCtrl ΔB1). Together, these data are consistent with the model that the *B12R* indel mutation in ΔB1mutB12 causes a loss of B12 function, leading us to infer that full length B12 is a repressor of ΔB1 growth.

**Fig 4:**
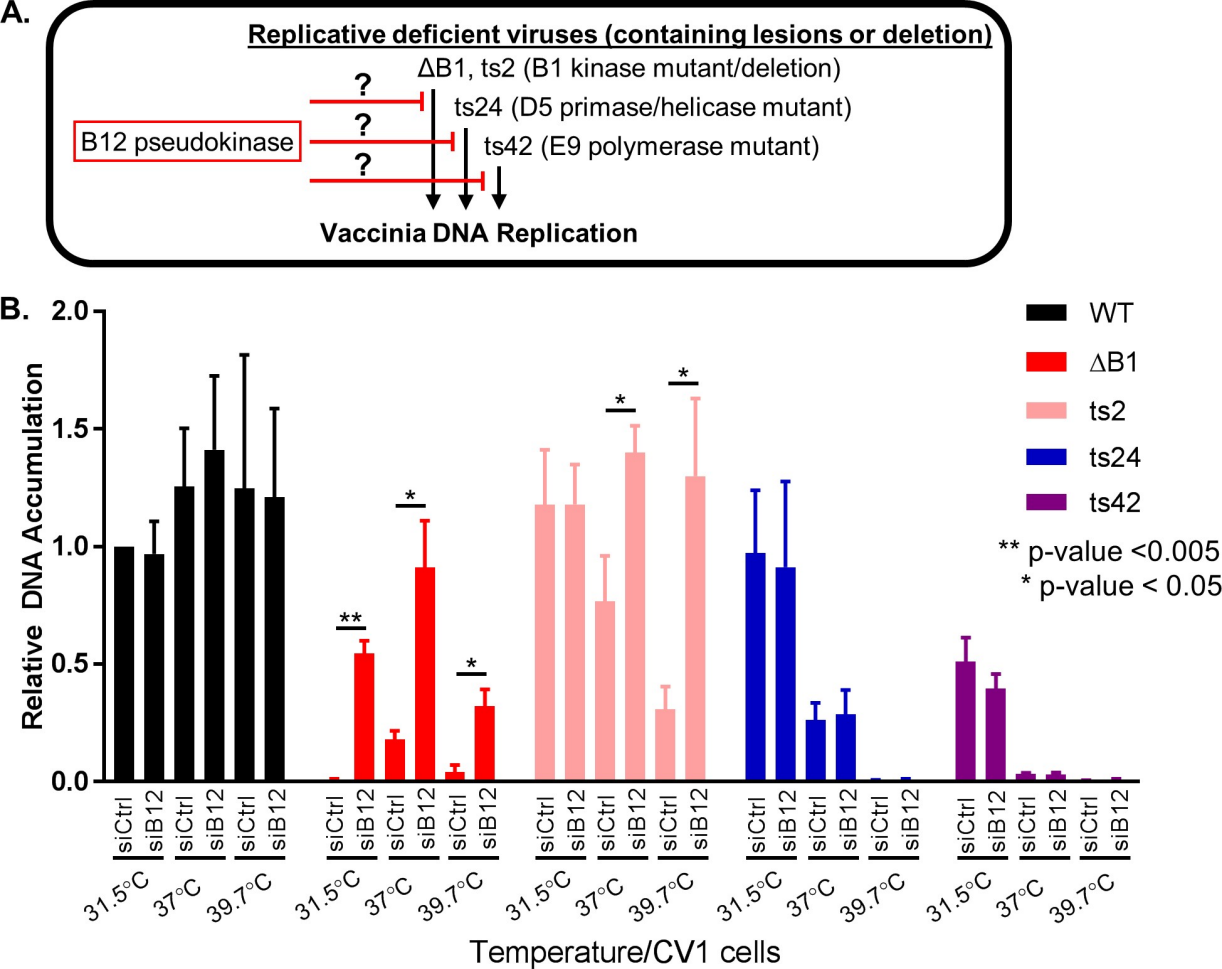
Rescue of DNA replication block using siB12 is specific for viruses lacking a functional B1. (A) Diagram of hypothesis that B12 is either a general inhibitor of DNA replication or specific to B1 kinase mutant viruses. (B) CV1 cells treated with siCtrl of siB12 were infected with WT (black), ΔB1 (red), ts2 B1 mutant (pink), ts24 D5 mutant (blue), or ts42 E9 mutant (purple) at a MOI of 3 and harvested 24h post infection for quantification of relative DNA accumulation. Infections were carried out at 31.5°C, 37°C or 39.7°C to provide permissive, semi-nonpermissive and nonpermissive temperatures respectively for the temperature sensitive mutant viruses.

It was interesting that siB12 treatment does not impact WT growth, but is only apparent in the absence of the B1 kinase, indicating that the B12 repressive function may not be active in the presence of B1. To explore this possibility further, we examined whether B12 depletion would increase DNA accumulation of other replication deficient vaccinia viruses, such as those with lesions in the D5 primase/helicase or E9 DNA polymerase. We posited that if B12 inhibition is directly linked to a B1 mediated pathway of promoting DNA replication, then depleting B12 will only rescue B1 mutant or deletion viruses (Fig 4A). Alternatively, depletion of B12 may also rescue growth of other replicative deficient viruses such as D5 or E9 mutant viruses, which would indicate that B12 inhibits DNA replication via a more general mechanism of action (Fig 4A). In order to determine which model fits B12 repressive function, we depleted B12 during infection with WT or mutant viruses and assayed for DNA accumulation. Viruses used for this assay included WT, ΔB1, a temperature sensitive B1 mutant (ts2) virus [5], a temperature sensitive D5 primase/helicase mutant (ts24) virus [44], and a temperature sensitive E9 DNA polymerase mutant (ts42) virus [45]. Infections were performed at permissive temperature (31.5°C), semi-permissive temperature (37°C), and nonpermissive temperature (39.7°C). DNA accumulation was quantified using qPCR during a synchronous infection of siCtrl or siB12 treated CV1 cells. The 24h DNA accumulation for WT virus remains constant at all three temperatures, independent of siB12 pre-treatment of cells (Fig 4B, black bars). The ΔB1 virus DNA accumulation was attenuated compared to WT in siCtrl cells as previously published [18]. Pre-treatment with siB12 rescues the ΔB1 DNA accumulation 58-fold, 5-fold, and >9-fold as compared to the siCtrl at 31.5°C, 37°C, and 39.7°C respectively (Fig 4B, red bars). The temperature sensitive ts2 B1 mutant virus follows a similar trend to the ΔB1 virus. Specifically, at non-permissive temperatures the siB12 treated cells have increased DNA accumulation 2-fold at 37°C and >4-fold at 39.7°C as compared to siCtrl cells for the ts2 virus (Fig 4B, pink bars). At permissive temperature the ts2 virus has similar DNA accumulation as the WT virus as expected. Importantly, the rescue in DNA accumulation observed for ΔB1 at all temperatures and for ts2 at non-permissive temperatures was not observed for the other two viruses with restricted DNA accumulation. Explicitly, the ts24 (blue bars) and ts42 (purple bars) viruses have similar restricted DNA accumulation at 37°C and 39.7°C temperatures for CV1 siCtrl cells compared to CV1 siB12 cells (Fig 4B). In summary, these data demonstrate that the depletion of B12 specifically rescues B1 mutant/deletion viruses, while B12 depletion does not enhance DNA replication for temperature sensitive D5 (ts24) and E9 (ts42) mutant viruses.

### Reconstitution of B12 in CV1 cells represses ΔB1 and ΔB1mutB12 replication

**Fig 5:**
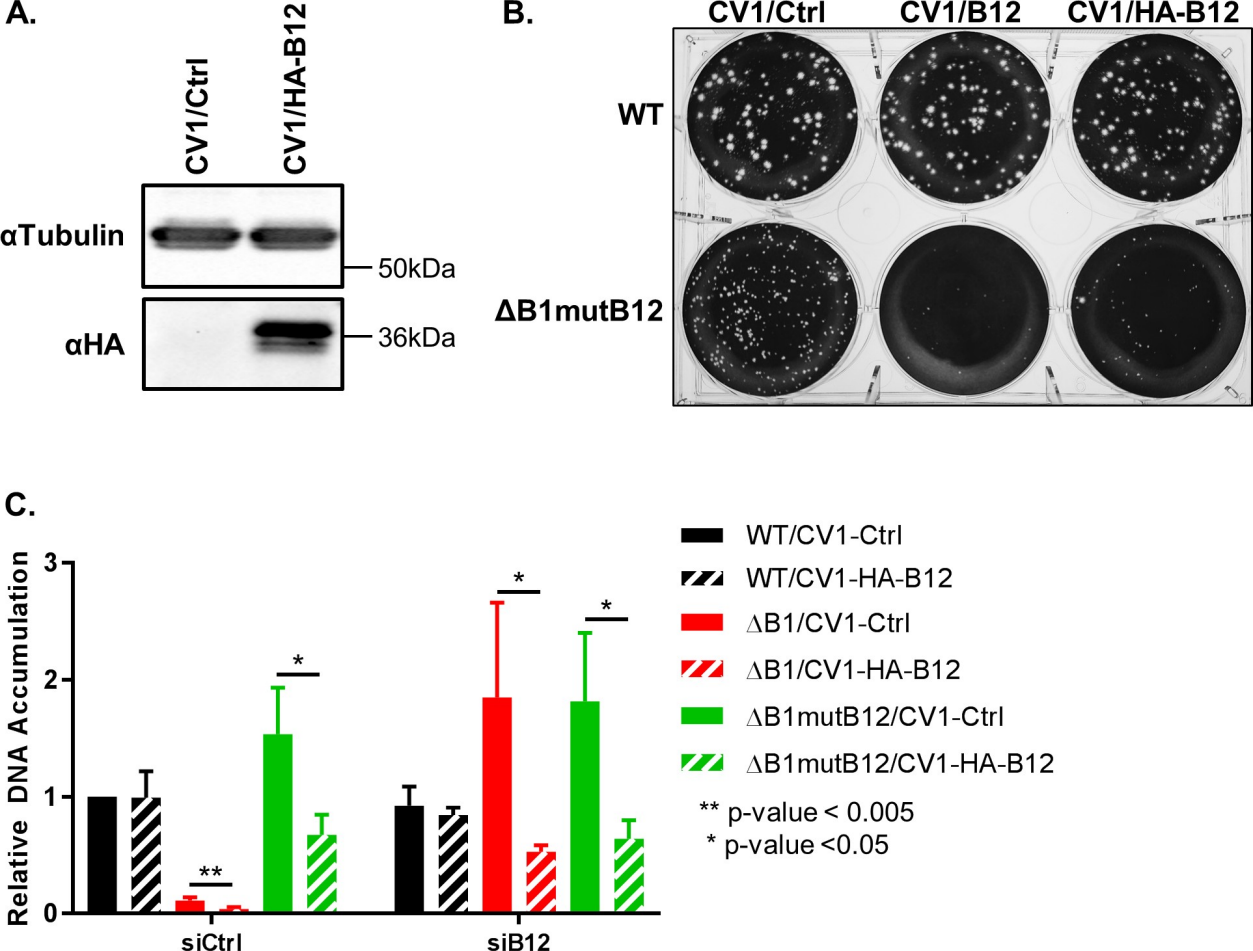
B12 reconstitution during infection with B1 and B12 naïve viruses repressed vaccinia replication. (A) Immunoblot analysis of control or HA-B12 (GeneArt) lentivirus transduced CV1 cells was completed to detect tubulin (loading control) and HA (HA-tagged B12). (B) CV1 control cells or cells stably expressing B12 or HA-tagged B12 were infected with WT or ΔB1mutB12-A3 at 300PFU/well and fixed 72h post infection. (C) 24h relative DNA accumulation quantification was completed for CV1 control or HA-B12 expressing cells transfected with siCtrl or siB12 and infected with WT (black), ΔB1 (red), or ΔB1mutB12-A3 (green) at a MOI of 3.

At this point we have shown that siRNA-directed loss of B12 rescues the ΔB1 and ts2 virus growth, specifically by increasing DNA replication. This supports a model in which B12 carries out a repressive function on DNA replication in the absence of a functional B1 kinase. To complement our above B12 depletion studies, we next hypothesized that expression of B12 from the cellular genome would be sufficient to inhibit replication of viruses lacking B1. To test this hypothesis, we began by generating cells stably expressing a HA-tagged or untagged codon optimized B12 (S4A Fig). Codon optimization for mammalian cells allows for enhanced expression of the gene by mammalian cells and in our system codon optimized B12 is resistant to the siB12 used to deplete B12 mRNA expressed by the virus. Expression of HA-B12 was confirmed using immunoblot analysis of whole cell lysates from both control and HA-B12 lentivirus transduced and selected CV1 cells (Fig 5A).

To test the repressive activity of reconstituted B12, we transfected control or HA-B12 expressing CV1 cells with siCtrl or siB12 for 24h and then infected cells with WT, ΔB1, or ΔB1mutB12 virus at a MOI of 3. The cells were harvested at 24h post infection and relative DNA accumulation of each vaccinia virus was quantified using qPCR. Using a combination of siB12 treatment during a ΔB1 infection and separately using the ΔB1mutB12 virus allows us to test B12 repressive activity via restoration on two different systems in which the viral B12 has been inactivated. First, we compared results from cells treated with the control siRNA. In cells transfected with siCtrl, the DNA accumulation for the WT virus is not altered by reconstitution of HA-B12 (Fig 5C, siCtrl, black solid and striped bars). In contrast, the ΔB1 virus exhibited a 2.7-fold reduction in relative DNA accumulation in CV1-HA-B12 cells as compared to CV1-Ctrl cells (Fig 5C, siCtrl, red solid and striped bars). Similarly, the relative DNA accumulation for ΔB1mutB12 virus was 2.3-fold lower in HA-B12 expressing cells than control cells (Fig 5C, siCtrl, green solid and striped bars).

Next, we compared cells in which viral B12 was depleted. In siB12 transfected cells, the WT virus DNA accumulation was not altered by HA-B12 expression in cells (Fig 5C, siB12, black solid and striped bars). Consistent with our model of B12 repressive activity during vaccinia virus infection in the absence of a functional B1 kinase, the DNA accumulation for the ΔB1 virus was 3.4-fold lower in HA-B12 expressing cells than control cells under siB12 conditions (Fig 5C, siB12, red solid and striped bars). Lastly, the ΔB1mutB12 replication in HA-B12 expressing cells was reduced 2.8-fold relative to control cells for siB12 transfected cells (Fig 5C, siB12, green solid and striped bars). Importantly, B12 expression from the cell was sufficient to inhibit DNA replication for both the ΔB1/siB12 and ΔB1mutB12 systems. These data further support a model in which B12 can downregulate vaccinia virus DNA accumulation in the absence of a functional B1 kinase.

Previously we demonstrated that depletion of B12 during a ΔB1 infection allows the virus to carry out productive infection as measured by the formation of plaques on CV1 cells (Figs 3C and S3E). To determine how reconstitution of wild-type B12 affects vaccinia productive infection, we carried out a plaque assay of either WT or ΔB1mutB12 infected control, B12 expressing, or HA-B12 expressing CV1 cells. Cells were fixed three days post infection. The WT virus plaque number and size was unchanged by the expression of B12 or HA-B12 in cells as compared to the control CV1 cells (Fig 5B, top row). Strikingly, after infection with the ΔB1mutB12 virus there was a consistent reduction in number and in size of plaques in both the B12 and HA-B12 expressing cells compared to control CV1 cells (Fig 5B, bottom row). Thus, the addition of the wild-type B12 decreases ΔB1mutB12 productive infection, providing additional evidence that B12 can impair poxvirus replication in the absence of B1.

### B12 is predominantly nuclear and solubilizes separate from chromatin-bound proteins

**Fig 6:**
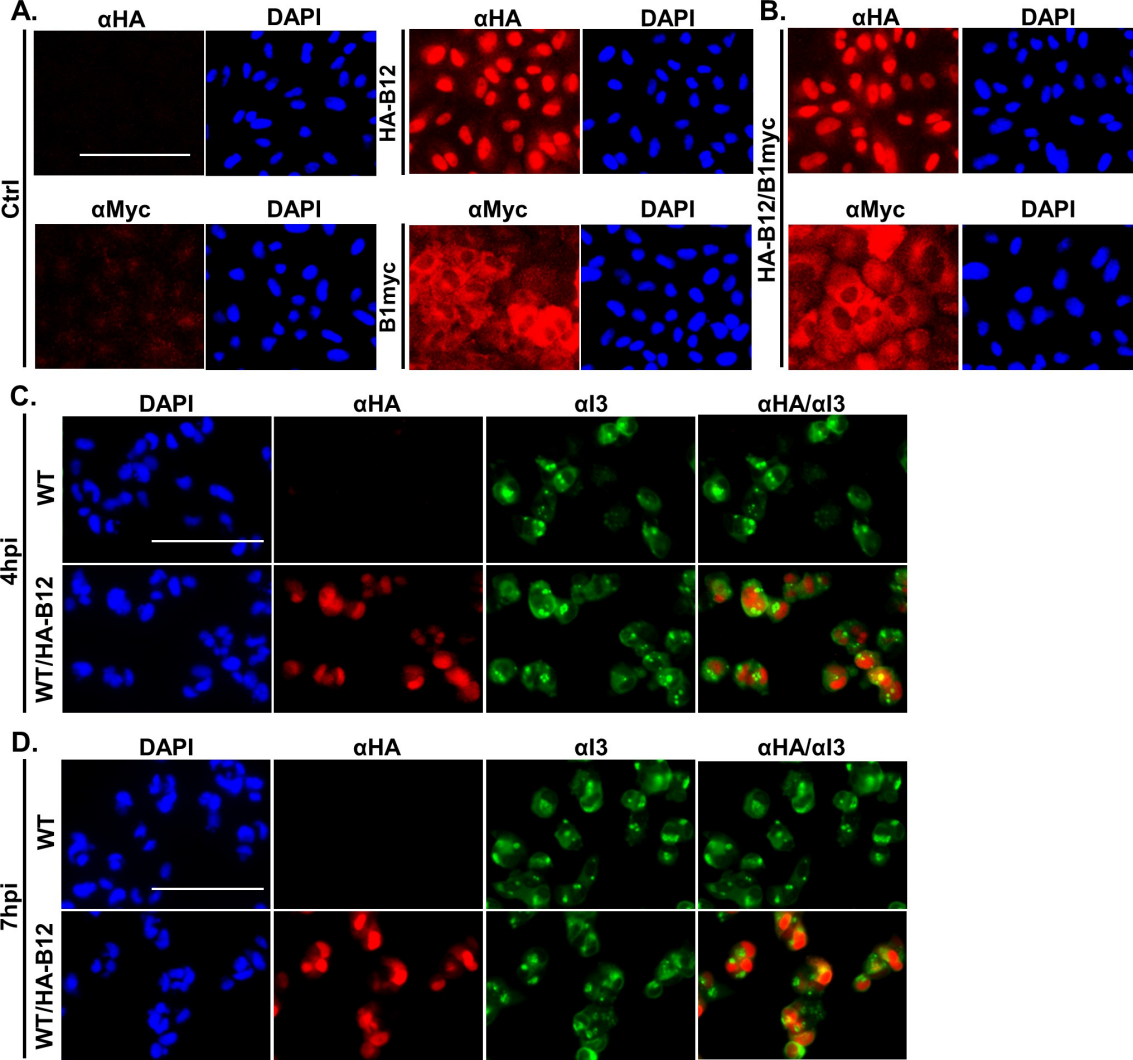
B12 exhibits a nuclear localization in uninfected and infected cells. (A) CV1 cells with or without HA-B12 (GenScript) mRNA transfection were used for immunofluorescence detection of HA-tagged B12 (red, top row). CV1 control and CV1-B1myc expressing cells were incubated with αmyc for B1myc detection (red, bottom row). All cells were stained with DAPI nuclear stain (blue). (B) B1myc expressing CV1 cells were also transfected with HA-B12 (GenScript) mRNA and separately incubated with a primary antibody to detect HA-tagged B12 (αHA, top red image) or myc-tagged B1 (αmyc, bottom red image) and DAPI (blue) nuclear stain. (C) CV1 cells were infected with WT or WT/HA-B12 virus at a MOI of 5 and fixed at 4hpi or (D) 7hpi for immunofluorescence analysis of HA-B12 detection (red), I3 ssDNA binding protein (green) and DAPI nuclear stain (blue). The scale bars represent 100μm.

To provide insight into the function of the B12 protein, we examined the subcellular localization of transiently expressed HA-tagged B12 (S4B Fig) in CV1 cells. One day after transfection, cells were fixed, incubated with αHA primary antibody with corresponding secondary antibody and stained with DAPI for immunofluorescence imaging of cells. The HA-B12 expressing cells show a clear nuclear localization as compared to control cells (Fig 6A, top row). By comparison, the B1 kinase localizes to the cytoplasm (Fig 6A, bottom row), as published previously [18]. Next, we tested whether B1 expression in cells could alter B12 subcellular localization by examining transiently expressed HA-B12 in cells expressing the myc-tagged B1 protein. The top panels show that HA-B12 still exhibits a nuclear localization in cells expressing the B1 kinase (Fig 6B, top row). Additionally, the B1 kinase does not have altered localization in the presence of HA-B12 expression and nuclear localization (Fig 6B, bottom row). Therefore, B1 expression does not detectably redirect B12 localization in this assay.

Although B12 localized to the nucleus in uninfected cells, it is possible that B12 localization is different during vaccinia infection. To address this question, homologous recombination was used to generate a WT/HA-B12 recombinant virus expressing the transgene from the nonessential viral TK locus. To assess HA-B12 localization during infection, CV1 cells were infected with WT or WT/HA-B12 virus at a MOI of 5 and fixed at either 4 or 7hpi, chosen to coincide with times of peak early gene expression and DNA replication. Interestingly, virus expressed HA-B12 exhibited a predominantly nuclear localization at 4 and 7hpi, (Fig 6C and 6D, αHA panel) similar to that observed in uninfected cells. Furthermore, HA-B12 nuclear localization was distinct from the viral, cytoplasmic replication factories as indicated by puncta formation of vaccinia I3 single-stranded DNA binding protein (Fig 6C and 6D, αI3 and αHA/αI3 panels).

**Fig 7:**
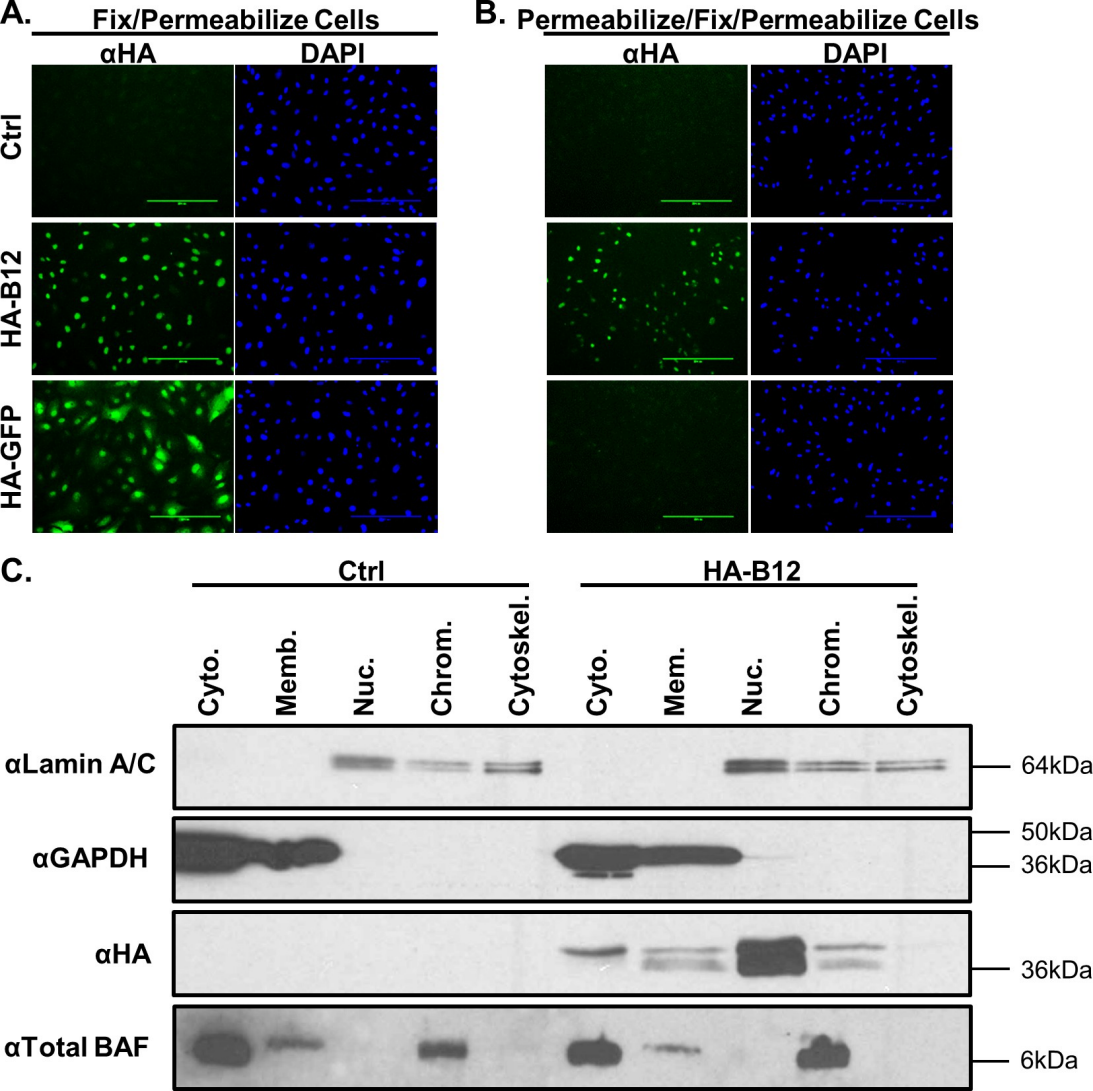
B12 nuclear localization is distinct from chromatin bound proteins. (A) CV1 cells were transfected with no mRNA, HA-GFP mRNA, or HA-B12 (GenScript) mRNA. Cells were either fixed then permeabilized to detect HA-tagged proteins or (B) prepermeabilized, fixed and then permeabilized again for detection of HA-tagged proteins remaining in cells following washes to remove unbound protein. The DAPI nuclear stain (blue) and αHA antibody (green) for detection of HA-GFP and HA-B12 were used. The scale bars in 7A and 7B represent 200μm. (C) Subcellular fractionation of CV1 control or HA-B12 (GeneArt) stably expressing cells was completed to separate cells into cytoplasmic extract (Cyto.), membrane extract (Memb.), soluble nuclear extract (Nuc.), chromatin-bound extract (Chrom.), and cytoskeleton extract (Cytoskel.). Lamin A/C, GAPDH and BAF protein detection were used as fractionation controls and HA was used to detect HA-tagged B12 protein.

To further characterize HA-B12 nuclear localization, we examined B12 solubility in two separate assays. First using an immunofluorescence based approach, we utilized a protocol in which cells are briefly treated with detergent prior to fixation to separate highly soluble proteins from those more strongly tethered to nucleic acids or cytoskeletal elements in the cell [46–52]. Cells transiently expressing HA-B12 or HA-GFP were either fixed then permeabilized or first prepermeabilized (0.1% Triton X-100), followed by fixing cells and a second permeabilization (0.2% Triton X-100) step. In CV1 cells fixed prior to permeabilization, the control cells (top row) had low background after αHA incubation, the HA-B12 (middle row) had a nuclear localization, and HA-GFP expressing cells (bottom row) showed diffuse localization of that protein (Fig 7A). For CV1 cells that were prepermeabilized, the αHA columns for control cells (top row) had low background, HA-B12 expressing cells (middle row) continued to exhibit bright, nuclear localization, while HA-GFP expressing cells (bottom row) exhibited only background levels of green fluorescence similar to the control cells (Fig 7B). In summary, pre-permeabilization of cells was sufficient to solubilize HA-GFP from cells while HA-B12 was retained in the nucleus under the same conditions. Together, these data suggest that B12 not only localizes to the nucleus of uninfected or infected cells, but also interacts with unknown binding partners there.

For comparison to the immunofluorescence based solubilization assay, we also examined the B12 fractionation profile following sequential treatment with a commercially optimized panel of extraction buffers. Following fractionation of control CV1 and CV1-HA-B12 cells, western blot analysis was performed using equal proportions of each fraction (Fig 7C). In addition to HA-B12, we examined the abundance of GAPDH, lamin A/C, and BAF in each fraction. As expected, GAPDH was most enriched in the cytoplasm with some also detected in the membrane fraction, but not in the other fractions. Lamin A/C was enriched in fractions expected to contain nuclear components and cytoskeleton. Previous studies have demonstrated that BAF is present both in the nucleus, where it binds chromatin, and free in the cytoplasm as summarized in a recent review article [53]. Our results here are consistent with those data and show BAF to be primarily present in a soluble form in the cytoplasmic fraction, and a chromatin-bound fraction. Interestingly, the HA-B12 protein was found primarily in the soluble nuclear fraction. Detectable HA-B12 was also present in the cytoplasmic, membrane and chromatin-bound extracts albeit at much lower levels. Together in concert with the immunofluorescence assays, these studies indicate that B12 localizes predominantly to the nucleus where it fractionates distinctly from BAF and likely other chromatin associated proteins.

### The ΔB1mutB12 virus is less sensitive to BAF antiviral activity than the ΔB1 virus correlating with altered BAF regulation

**Fig 8:**
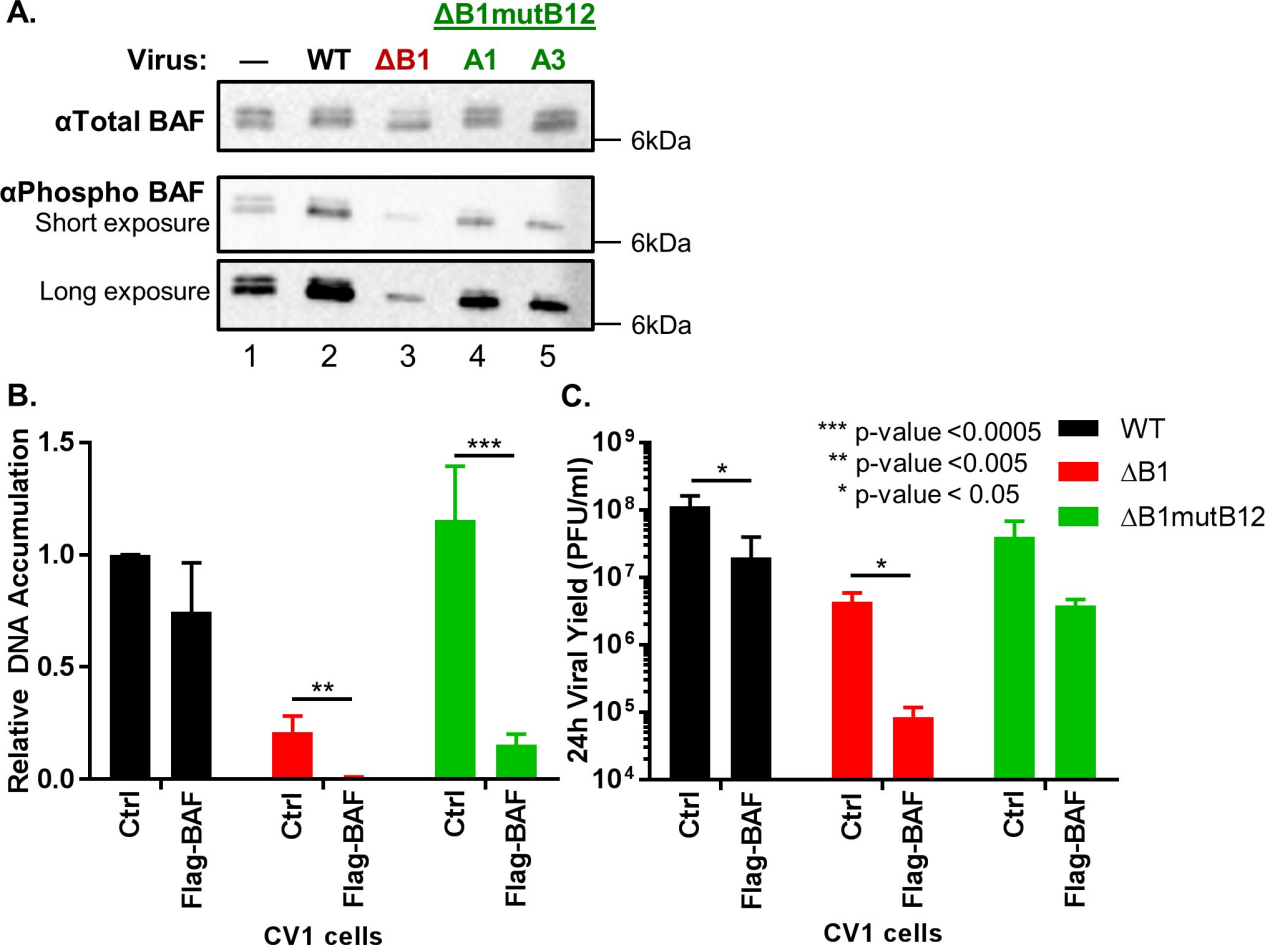
The ΔB1mutB12 virus restriction of BAF antiviral activity is greater than the ΔB1 virus. (A) Immunoblot analysis of total BAF protein (top panel) or phosphorylated BAF (bottom panel) in CV1 cells uninfected or infected with WT, ΔB1, ΔB1mutB12-A1, or ΔB1mutB12-A3 at a MOI of 10. Cells were collected at 6h post infection. (B) Control or CV1 cells expressing 3XFlag-tagged BAF in excess were infected with WT (black), ΔB1 (red), or ΔB1mutB12 (green) at a MOI of 3 and harvested at 24h post infection for analysis of relative DNA accumulation or (C) viral yield titration on CV1-B1myc cells.

The vaccinia virus B1 kinase regulates the antiviral protein BAF via phosphorylation of its N-terminus, which inactivates BAF binding to dsDNA [54] and repression of vaccinia virus DNA replication [6]. To investigate whether a link exists between BAF and the rescued growth phenotype of ΔB1mutB12, we measured both phosphorylated BAF levels during ΔB1mutB12 infection and DNA replication of the ΔB1mutB12 virus in cells overexpressing BAF. First for immunoblot analysis of BAF, CV1 cells were infected with WT, ΔB1, and ΔB1mutB12 viruses at a MOI of 10. Infected cells were harvested at 6h post infection along with an uninfected control sample, lysed in the presence of phosphatase and protease inhibitors and subjected to immunoblot analysis. BAF specific antibodies recognizing either total BAF (phospho-BAF upper band and unphosphorylated BAF lower band) or only phosphorylated BAF were used to detect protein levels under each condition. The total BAF levels were similar between uninfected and each infected sample in multiple experiments (Fig 8A, top row). Regarding phosphorylated BAF levels, lysates from WT infected cells contained increased levels of modified BAF as compared to the uninfected control (Fig 8A, αPhospho BAF, compare lanes 1 and 2). In contrast, the ΔB1 infected cells show a consistent reduction in phospho-BAF levels as compared to both uninfected and WT infected cells (Fig 8A, αPhospho BAF, compare lane 3 with lanes 1 and 2). Surprisingly, the ΔB1mutB12-A1 and ΔB1mutB12-A3 viruses had phospho-BAF amounts that were repeatedly higher than the ΔB1 infected cells (Fig 8A, αPhospho BAF, compare lanes 4 and 5 with lane 3), but not to the same level as those in WT infected lysates. Consistent with this representative immunoblot, infection with ΔB1mutB12-A1 or ΔB1mutB12-A3 virus clearly correlates with elevated phosphorylated BAF as compared to ΔB1 infected cells in multiple biological replicates (S5A–S5D Fig). This suggests that the absence of a functional B12 protein during ΔB1 infection correlates with increased BAF phosphorylation and consequently may affect BAF’s antiviral activity.

The intermediate level of BAF modification in ΔB1mutB12 lysates compared to WT and ΔB1 infected lysates may impact BAF’s capacity to block viral DNA replication. If true, one would predict that the ΔB1mutB12 virus may remain sensitive to BAF levels, but to a lesser degree than the ΔB1 virus. To test this model, cells stably overexpressing 3XFlag-tagged BAF protein (~10–12 fold increased BAF protein as compared to endogenous BAF levels) or control cells were infected with WT, ΔB1, or ΔB1mutB12-A3 virus at a MOI of 3 and levels of DNA accumulation measured. The DNA accumulation for WT, ΔB1, and ΔB1mutB12-A3 infected cells was reduced by 1.3-fold, 26-fold, and 7.5-fold respectively in Flag-BAF cells as compared to control CV1 cells (Fig 8B). This data demonstrates a significant attenuation of both ΔB1 and ΔB1mutB12 DNA accumulation, but not WT virus, when BAF levels are increased. Furthermore, the viral yield of WT, ΔB1, and ΔB1mutB12-A3 viruses were 5.8-fold, >51-fold and >10-fold reduced in Flag-BAF cells than control CV1 cells (Fig 8C). It is interesting to note that in the presence of increased BAF, the ΔB1mutB12 DNA accumulation and viral yield levels are 19-fold and 45-fold higher respectively than the ΔB1 virus (Fig 8B and 8C, compare green bars to red bars from cells expressing Flag-BAF). Combined, these assays demonstrate a correlation between loss of B12 function and increased phosphorylated BAF levels, and support the conclusion that ΔB1mutB12 virus represents an intermediate sensitivity to BAF’s antiviral activity as compared to the ΔB1 and WT viruses.

## Discussion

A growing body of evidence indicates that vaccinia kinases modulate multiple signaling pathways, although which of these are consequential for viral fitness is less well understood [55–60]. A primary function of B1 is to inactivate the host defense activity of BAF and allow DNA replication to proceed [6]. Notably, recent studies describe an incomplete rescue of a B1 deletion virus following shRNA-mediated depletion of BAF, suggesting that additional important functions for B1 exist [18]. To expand our current understanding of B1 driven signaling we subjected the B1 deletion virus to experimental evolution. When coupled with whole genome sequencing, this allowed us to identify a vaccinia mutation correlating with marked suppression of the fitness defect caused by the deletion of the *B1R* gene. Experiments using this approach to investigate mechanisms of poxvirus adaption after deletion of other genes have been performed previously, revealing that these pathogens can undergo rapid genetic expansions to form an ‘accordion’ of copies of a compensating gene to enhance virus production [26, 27]. Other adaptation studies have demonstrated that alterations in a small number of amino acids may be sufficient to detectably compensate for the loss of a gene [28, 29]. In contrast to these examples exploiting gain of function mutations to improve the fitness of a mutant virus, the data presented here indicate that a divergent mechanism involving the rapid disruption of a suppressor gene, *B12R*, is sufficient for enhancement of ΔB1 virus replication.

The initial evidence of a link between B1 and the mutation of *B12R* was quite compelling, as greater than 48% of the read counts included an indel at the identical nucleotide site in two independently adapted ΔB1 viruses. The evidence that B12 mutation is linked to viral adaptation was further supported through targeted Sanger sequencing of DNA isolated from individual plaques from all three adapted viral stocks. A fascinating aspect of the major indel mutation is its location in a homopolymeric run of eight adenines. Indeed, each of the less prevalent indel sites mapped via Sanger sequencing was also within a homopolymeric run of 4–5 nucleotides. Such sites of repeated sequence may cause polymerase stuttering and favor indel introduction, perhaps contributing to how quickly the virus was able to adapt in our assay [61–64]. Thus, independent of our goal to discover novel interactions between B1 and other vaccinia genes, this study is insightful as an experimental model of reductive evolution during poxvirus adaptation. Indeed, rigorous sequence comparisons of gene maps within members of the Orthopoxvirus genus have led to the theory that gene loss has played a defining role in adapting family members to specific hosts [30]. Notably, these bioinformatics analyses predicted that indels introduced at simple sequence repeats within viral ORFs are likely a common molecular mechanism for gene fragmentation [30]. Our data now provide strong experimental evidence that indel introduction can indeed occur rapidly at homopolymeric sequence repeats during viral replication, causing gene loss. Of further significance, it is clear that reductive evolution can lead to substantial fitness gains for these pathogens and may be a stronger selective pressure on viruses than previously appreciated.

Upon discovering the frame-shifting indel present within the adapted viruses, we posited that the truncation of B12 may enhance viral fitness either via a loss of B12 function or a gain of function, the latter scenario possible if the truncation removed a theoretical autoinhibitory domain from the protein. Experiments employing siRNA to deplete B12 led to three important observations, allowing these two possibilities to be distinguished. First, in the presence of siB12, the replication of ΔB1 virus DNA and yield of progeny virus increased to levels very similar to that observed with the adapted ΔB1mutB12 virus in most cell lines tested. This outcome supports the loss of B12 function scenario. Second, while siB12 treatment led to the same decrease in B12 mRNA from the ΔB1 virus and the ΔB1mutB12 virus, there was no reduction in DNA accumulation or viral yield from the ΔB1mutB12 virus with siB12 treatment arguing against a gain of function mutation. Third, while siB12 enhanced the replication of the temperature sensitive ts2 B1 mutant virus, it did not affect the WT virus or other mutant viruses that exhibited reduced DNA replication at less permissive temperatures because of defects in the vaccinia polymerase or primase/helicase proteins. This third point emphasizes that the repressive function of B12 is controlled by the B1 kinase. Together, these assays demonstrate that loss of B12 can suppress the fitness defects of B1 mutant viruses, but does not enhance replication of viruses containing a wild-type *B1R* gene. These genetic data are consistent with a model in which the B12 pseudokinase is capable of acting as a repressor of vaccinia replication in a B1-dependent manner. The inference that B12 function is masked by B1 is also consistent with previous single gene knockout studies of B12. Specifically, thorough examination of a virus lacking the majority of the *B12R* gene revealed no detectable change in viral fitness in cell culture or mouse pathology as compared to WT virus controls [41, 42]. Those results led to B12 being designated as one of the nonessential genes of vaccinia, which is also supported by the fact that although the *B12R* gene is present in all members of the Orthopoxvirus genus, the closely related taterpoxvirus and variola virus have a nonsense or deletion mutation, respectively, in their B12 homolog [61]. However, our studies demonstrate that while nonessential, B12 pseudokinase is not without function. Furthermore, our work adds to a growing body of evidence indicating that poxvirus genes categorized as nonessential in tissue culture and *in vivo* studies based on single gene deletions should be investigated in multigenic knockout backgrounds, especially if they belong to gene families [65].

To understand the possible ramifications of our B1/B12 model we find it informative to draw from virology as well as potentially analogous systems in the broader scientific literature. For example, the signaling relationship exhibited by B1 and B12 demonstrates similarity to features of toxin-antitoxin (TA) systems common in bacteria or poison-antidote modules more recently uncovered in higher organisms [34–37, 39, 40, 66–68]. While TA systems proceed via diverse and often poorly understood molecular mechanisms, they are generally comprised of two genes, one of which is capable of decreasing the overall fitness of the organism and is referred to as the toxin. Critically however, the toxin’s repressive activity is inhibited in cells expressing a cognate antitoxin gene product. In some instances, the TA system is regulated by upstream signals that can influence antitoxin stability and/or activity, thus potentially benefiting an organism by slowing its growth in response to stress [34, 35, 37, 69]. However, in other examples, TA modules provide little or no known benefit for their host, instead behaving as a type of ‘selfish’ genetic element, perhaps to ensure their conservation in an organism by addicting the organism to an antidote against the TA encoded ‘poison’ protein [38–40]. As described herein, some attributes of toxin-antidote genetic elements also apply to the B1 and B12 pair. Consideration of parallels between B1/B12 signaling and TA systems has implications beyond poxviruses; this viral kinase and pseudokinase exhibit high sequence similarity to a family of mammalian proteins known as the VRKs (vaccinia related kinases) also containing kinase and pseudokinase domains [7, 9, 17]. Thus, it is conceivable that the mammalian VRK family members and possibly some of the other numerous kinase-pseudokinase pairs in nature exhibit features of TA modules as well.

**Fig 9:**
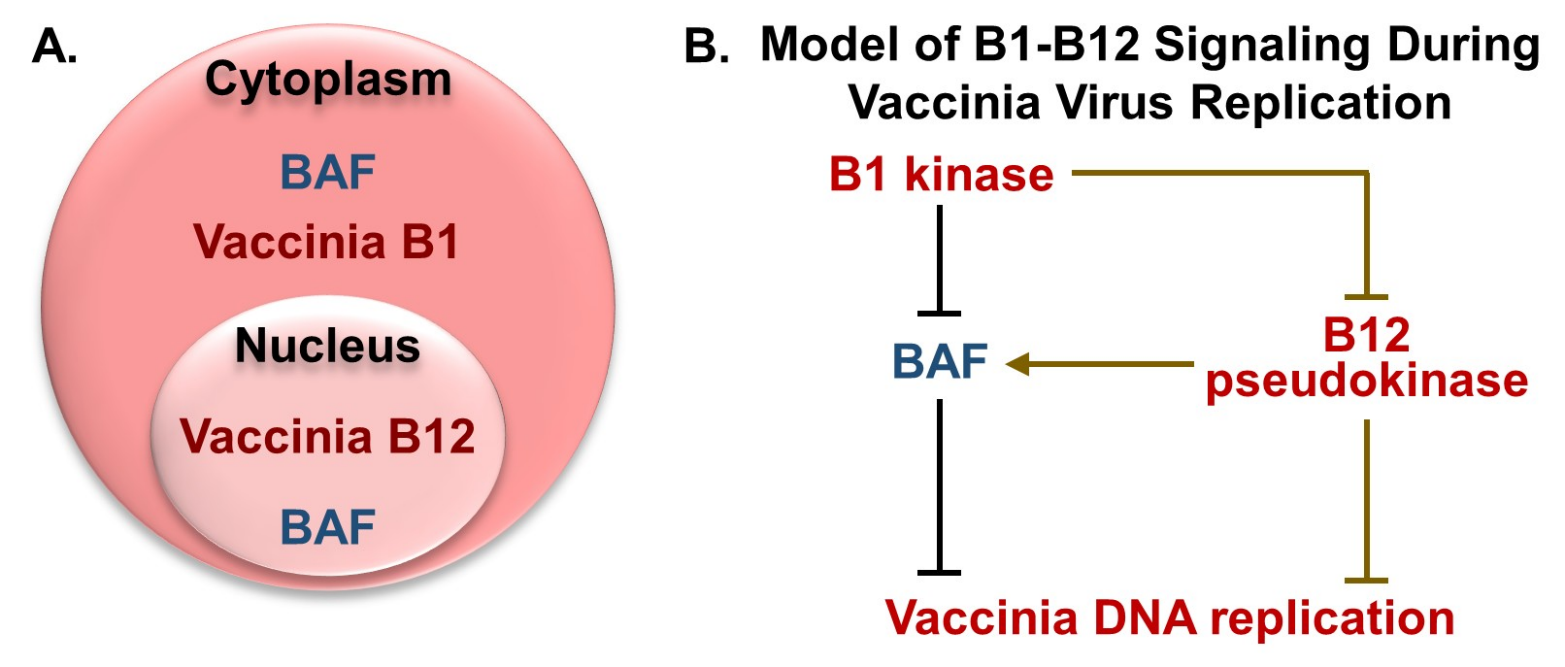
Subcellular localization of B12 and working model of B1/B12/BAF signaling during vaccinia infection. (A) Vaccinia B1 kinase overlaps with cytoplasmic localized host BAF protein whereas vaccinia B12 pseudokinase shares a nuclear subcellular localization with the nuclear fraction of BAF. (B) The B1 kinase participates in restriction of BAF’s antiviral function against vaccinia DNA replication in the cytoplasm, while also repressing B12 negative regulation of vaccinia DNA replication through an unknown mechanism that is partly mediated via BAF regulation. Direct interactions and/or signaling through additional factors may be required for B1-B12 signaling and B12-BAF signaling, and are depicted using gold lines. B1-BAF interaction and BAF binding to dsDNA are direct interactions and denoted in black lines.

Returning to our data of B12 repressive activity, we have begun to dissect the B12 mechanism of action. Due to the apparent antagonism of B12-mediated repression in the presence of B1, we were interested in examining where B12 localized in cells with respect to the B1 kinase. We hypothesized that B12 may be localized to the cytoplasm where the B1 kinase is found in both uninfected and infected cells [16, 18]. However, we were somewhat surprised to find that B12 is found predominately in the cell nucleus, even in cells expressing the B1 kinase in the cytoplasm and in vaccinia infected cells (Fig 9A). Solubilization studies suggest that B12 is tethered to an unknown partner protein in the nucleus; it is tempting to hypothesize that identifying this partner may provide a clue as to B12’s mechanism of action.

We can only speculate as to the function of B12 in the nucleus at this time. Other poxviral proteins including C4 [70], C6 [71], C16 [72], B14 [73], K7 [74], N2 [75], F16 [76], and E3 [77] can be detected in the nucleus and have been found to impact innate immune signaling in most cases. However, there is no precedent to date for a nuclear poxviral protein affecting viral DNA replication. Intriguingly, the B12 connection to B1 may also partly incorporate BAF (Fig 9B). This B1-regulated antiviral host factor is prevalent in both the nucleus and cytoplasm (Fig 9A). For this reason, we investigated how the presence of B12 affects the ability of BAF to act as a host defense against vaccinia. Measurement of phosphorylated BAF levels in infected cells demonstrated that WT virus led to a clear increase in BAF phosphorylation when compared to ΔB1 infected cells, as has been previously published [18]. Interestingly, BAF phosphorylation in ΔB1mutB12 infected cells was greater than in ΔB1 infected cells, albeit not to the same levels as during WT virus infection. Parallel studies of viral yield in cells overexpressing BAF demonstrated that inhibition by BAF was strongest on the ΔB1 virus and, while still observed during ΔB1mutB12 infection, BAF affected this adapted virus to a lesser degree. These data suggest a model in which B12 functions via a BAF dependent mechanism, albeit only in part (Fig 9B). Specifically, our previously published data that BAF-depletion only modestly rescues the ΔB1 phenotype [18] leads us to propose that B12 is also working through a distinct and BAF-independent pathway as shown in Fig 9B. It is provocative to consider that this pathway may be executed or triggered by B12 from within the nucleus, and will require further study for validation.

In conclusion, our study of the vaccinia B1 kinase has yielded numerous unexpected insights into poxvirus adaptation pathways and signal transduction circuitry. Our results evince that B1 contributes to viral fitness via antagonism of BAF and B12 proteins, and raise new questions regarding the underlying mechanism of action for this pseudokinase and how it is governed by B1. Future investigation of whether the inhibitory action of B12 extends to other poxviruses including those lacking a B1 kinase, and perhaps even other pathogens, will be particularly intriguing. Finally, as pseudokinase domains are prevalent in diverse eukaryotic organisms [32, 33], such studies will likely be of broad biological interest.

## Methods

### Cell culture

African green monkey kidney CV1 cells were purchased from Invitrogen Life Technologies. Human cervix epithelial adenocarcinoma HeLa, human lung epithelial carcinoma A549, mouse fibroblast L929, and human thymidine kinase-negative 143B osteosarcoma TK(-) cells were obtained from ATCC. CV1, HeLa, L929, and 143B TK(-) cell lines were maintained in the Dulbecco’s modified Eagle’s media (DMEM) supplemented with 10% fetal bovine serum (FBS; Atlanta Biologicals), 100 Units/ml penicillin (Life Technologies), and 100 μg/ml streptomycin (Life Technologies) in 5% CO_2_ at 37°C. The A549 cells were maintained in DMEM/Ham’s F-12 nutrient mixture (1:1) medium supplemented with 10% FBS and penicillin-streptomycin following identical conditions as stated above.

### Generation of stably transduced cell lines

The lentiviruses encapsidating pHAGE-HYG-MCS [78] (control), pHAGE-HYG-B1myc [18], and pHAGE-HYG-3XFlag-BAF (plasmid was generously provided by Dr. Paula Traktman) were used to stably transduce cells as previously described [18, 79]. Alternatively, a two plasmid helper system was used to generate lentiviruses encapsidating pHAGE-HYG-MCS (control) or pHAGE-HYG-B1myc. The two plasmid helper system included pVSVG and psPAX2, a gift from Didier Trono (Addgene plasmid # 12260), was combined with the transfer plasmid for transfection of 293T cells. Fresh media supplemented with 5mM sodium butyrate (EMD Millipore Corp.) was added to cells 16h post transfection. At 24h post transfection, fresh media supplemented with 10mM HEPES (Fisher Scientific) was added to the cells. Virus in supernatant was collected at 48h post transfection, polybrene (Fisher Scientific) was added to lentivirus at 10μg/ml, and stocks were stored at -80°C. Transduced CV1 cells were selected with 200 μg/ml hygromycin B (Invitrogen).

The lentivirus generation and stable expression of HA-tagged and untagged B12 cells used the pHAGE-HYG-MCS-HA-B12 or pHAGE-HYG-MCS-B12 construct which were produced by PCR amplifying a codon-optimized HA-B12 ORF in the pcDNA3.1 vector purchased from GeneArt (S4A Fig). The primers used for PCR amplification are found in S2 Table. HA-B12 or B12 ORF was then cloned into the BamHI site within the pHAGE-HYG-MCS multiple cloning site. Lentivirus generation used the four plasmid helper system (pVSVG, pTat, pREV, pGag/Pol), following the same protocol summarized above for transduction of CV1 cells.

### WT/HA-B12 recombinant virus generation

The recombinant WT/HA-B12 virus expresses an additional vaccinia *B12R* gene with a 5’ HA epitope sequence from the nonessential, thymidine kinase (TK) locus. This virus was generated by homologous recombination using standard protocols and pJS4 variant kindly shared by Paula Traktman laboratory [6]. Briefly, the HA-B12 sequence from the virus was amplified using F and R primers containing XhoI or NheI restriction sites respectively (S2 Table) and cloned into a pJS4 variant [80] flanking it with regions homologous to the vaccinia TK gene. Next, CV1-B1myc cells were infected with WT virus at MOI = 0.03 followed by transfection 3hpi with 3 μg linear pJS4-HA-B12 per 35mm well. Cells were harvested 48hpi, freeze/thawed three times, and used for virus titrations on CV1-B1myc cells. Recombinant viruses went through two rounds of purification by infecting 143B TK(-)/B1myc cells (lacking cellular expression of thymidine kinase) and treatment with 25 μg/μl bromodeoxyuridine (BrdU) to reduce productive infection of WT virus with an intact TK locus (control infections were completed without BrdU selection). WT/HA-B12 viruses were plaque purified three times and confirmed to be a pure stock using PCR amplification of viral DNA and immunofluorescence detection of HA-B12 protein in 50/50 plaques. An expanded preparation of this virus from a freeze/thawed lysate of infected CV1-B1myc cells was used for immunofluorescence assays.

### Viruses and viral infection assays

Viruses used for experiments include the following: wild-type (WT), B1 deletion (ΔB1) [18], ΔB1mutB12-A1, ΔB1mutB12-A2, ΔB1mutB12-A3, B1-mutant Cts2 [5], D5-mutant Cts24 [44], E9-mutant Cts42 [45], and WT/HA-B12 WR strain vaccinia viruses. These viruses were expanded on either BSC40, CV1 or CV1-B1myc cells and purified using a sucrose cushion. The ΔB1mutB12-A1, -A2, -A3 viruses were generated by infecting CV1 cells at a MOI of 0.1 in three independent 10cm plates. Virus was propagated in cells two days at 37°C before cell harvest. Cells were pelleted and resuspended in 1ml PBS. 100μl cells in PBS were saved for DNA purification and remaining cells were pelleted and resuspended in 900μl 10mM Tris pH 9.0 for virus titration. After freeze/thawing three times, the three independent virus stocks were titrated on B1myc expressing CV1 cells. Using these titers for passage 1 viruses, 10cm plates of CV1 cells were infected at a MOI of 0.1 and allowed to propagate on cells for two days at 37°C. Serial passage of viruses in CV1 cells at a MOI of 0.1 was completed for 7 total passages with either two or three days of propagation before cell harvest. Each passage of virus was titered on complementing, B1myc expressing CV1 cells.

Plaque assays were completed using either 200 or 300 plaque forming units (PFU) per well. For the ΔB1 adapted virus plaque assays, control or B1myc expressing CV1 cells were infected with WT, ΔB1, or ΔB1 adapted virus A1 for passages 1 through 7. Cells were fixed and stained at 72h post infection. The plaque assay of B12 depletion during WT, ΔB1, and ΔB1mutB12-A3 infection was completed by infecting cells 24h post transfection with siRNA. 72h post infection cells were fixed and stained. The plaque assay on CV1 control or HA-B12 stably expressing cells were fixed 72h post infection with WT or ΔB1mutB12-A3 virus.

Viral growth assays were conducted in multiple cell lines. One-step 24h viral growth assays were completed by infecting a monolayer of CV1, HeLa, A549, L929, or transduced CV1 cells with WT, ΔB1, ΔB1mutB12-A1, or ΔB1mutB12-A3 virus at a MOI of 3 and incubated at 37°C. 24h post infection cells were harvested for downstream DNA accumulation and viral yield quantification. Half of the cells harvested were pelleted and resuspended in PBS for DNA purification and qPCR while the other half was resuspended in 10mM Tris pH 9.0, freeze/thawed three times, and serially diluted for titration on CV1-B1myc cells. For one-step growth assays with siRNA treated cells, CV1 cells were infected 24h post transfection with siRNA. For experiments with temperature-sensitive mutant viruses, infections were carried out at 31.5°C, 37°C, and 39.7°C. Viral growth was also measured at multiple time points for CV1 cells infected with WT, ΔB1, or ΔB1mutB12-A3 virus. Cells were infected with a MOI of 3 and harvested at 3, 7, 16, and 24h post infection and used for both DNA accumulation and viral yield quantification. For multi-step growth curves, CV1 cells were infected at a MOI of 0.01 and harvested at 48h post infection for viral yield measurement by titration of samples on CV1-B1myc cells. Multi-step growth assay in siRNA treated cells were carried out at 24h post transfection, with cell harvests at both 7 and 48h post infection for viral yield quantitation.

WT virus was used for infections of cells transfected with pJS4 plasmid constructs. CV1 cells were infected at either a MOI of 3 or 5 and harvested 24h post infection for immunoblot analysis of HA-B12wt and HA-B12ΔA690 expressed from the pJS4 vector late viral promoter.

For detection of early gene expression, CV1 cells were infected with WT, ΔB1, or ΔB1mutB12-A3 at a MOI of 3 and harvested 4h post infection for RNA extraction from cells.

In the BAF immunoblot assay, CV1 cells were either uninfected as a control or infected with WT, ΔB1, ΔB1mutB12-A1, or ΔB1mutB12-A3 virus at a MOI of 10 to ensure synchronous infection. Cells were harvested at 6h post infection and lysates were subjected to immunoblot analysis.

### Sequencing

For complete genome sequencing of the WT (from the Wiebe laboratory), ΔB1, ΔB1mutB12-A1, and ΔB1mutB12-A3 viruses 1 ng of viral DNA from each sample was used to construct sequencing libraries. Libraries were constructed using the Nextera XT kit from Illumina per manufacturers suggestions. An aliquot of the resultant multiplexed library of four viral isolates was sequenced on the MiSeq V2 instrument. 150 base pair (bp) paired-end sequencing was performed. The paired reads of 150 bp (trimmed when necessary to remove adaptors and ends of reads with lower QC scores) was provided. Next, Illumina paired-end sequence reads were filtered using the program fastq_quality_filter from FASTX-Toolkit 0.0.14. The read pairs with at least 90% bases having quality of 30 were used to map to reference genome of Vaccinia virus WR (reference genome NC_006998.1). Bowtie2 version 2.2.4 was used for accurate and efficient mapping. Sequence data was uploaded to SRA database (PRJNA490542). The estimated overall coverage of each of the samples (using only the high quality paired reads) is between 800 and 2000x based on a genome size of 220kb.

Sequence data was analyzed for gene duplications, point mutations and insertion or deletion (indel) mutations within protein coding regions of the genome using a self-developed pipeline including samtools/bcftools. Sequence discrepancies that occurred in <5% of the read counts for a single nucleotide call were not included in further analysis. The mapped reads were visualized using Integrated Genome Browser (IGV 2.3.59). Complete genome sequences were aligned for all sequenced viruses and compared to the WT (Wiebe) virus to identify gene duplications. Point mutations were assessed by comparing WT (Wiebe) to WT WR (reference genome NC_006998.1) in the NCBI database, ΔB1 to WT (Wiebe), and both ΔB1-A1 and ΔB1-A3 to ΔB1 sequenced genome. Lastly, indel mutations were discovered by comparing the indel changes between WT (Wiebe) and WT WR (reference sequence) with the change in indel mutations for ΔB1, ΔB1-A1, or ΔB1-A3 and WT (Wiebe) as in Fig 1B or by alignment the whole genome sequence for ΔB1-A3 to the WT WR (reference sequence) genome in Fig 1C. Mutations in greater than 5% of the read counts at a single nucleotide position were considered significant mutations in the mixed population of ΔB1-A1 and ΔB1-A3 viruses.

For *B12R* targeted Sanger sequencing, ΔB1mutB12 virus lineages A1, A2, and A3 were plaque purified twice on CV1 cells. Virus was expanded on CV1 cells and DNA was purified from the resultant viruses using a GeneJET whole-blood genomic DNA purification minikit (Thermo Scientific). Purified DNA samples were subjected to Taq based PCR using 1μM each B11R F and B13R R primers (S2 Table). Following PCR amplification, B11-B13 products were cleaned using a QIAquick PCR purification kit (Qiagen). PCR products were then submitted for Sanger DNA sequencing (S2 Table) and analyzed for lesions within *B12R*.

### DNA/RNA purification and qPCR

For fold DNA abundance quantified for total VACV DNA and the *B1R* gene specifically, DNA was extracted from WT, ΔB1, ΔB1 adapted viruses A1 passages 1–7, ΔB1 adapted viruses A2 passages 1–7, and ΔB1 adapted viruses A3 passages 1–7 infected CV1 cells (for infection details see section “Viruses and viral infection assays”). The WT and ΔB1 control samples came from one-step infection DNA samples in CV1 cells. DNA was purified using a GeneJET whole-blood genomic DNA purification minikit (Thermo Scientific). The Bio-Rad iTaq Universal SYBRGreen supermix was used with quantitative polymerase chain reaction (qPCR) as previously described [81] with the addition of a *B1R* specific primer set. In brief, the WT purified DNA sample was serially diluted to generate a standard curve and determine amplification efficiency of *HA* (total VACV DNA) and *B1R* primer sets (S2 Table). For WT and ΔB1 controls about 10ng DNA and 1μM primers were combined in a single reaction. Variable amounts of DNA were used for ΔB1-A1, ΔB1-A2, and ΔB1-A3 passages 1–7, although the volume used was constant when combined with 1μM primers per reaction.

The one-step 24h viral DNA accumulation samples were treated similarly to the DNA extraction and purification above. The infection protocol is detailed under one-step viral growth infections in methods section “Viruses and viral infection assays”. Samples were subjected to qPCR with the *HA* specific primer set (S2 Table) in triplicate to determine relative viral DNA accumulation.

Early viral gene expression was determined by infecting CV1 cells as detailed in methods “Viruses and viral infection assays” section and harvested at 4h post infection. RNA was extracted from cells using the RNeasy Mini Kit (Qiagen). Reverse transcription of RNA into cDNA was carried out using a high-capacity cDNA reverse transcription kit (Thermo Fisher Scientific, Applied Biosystems). Then using probe and primer sets specific for either B12 or B13 cDNA, qPCR was used to quantify relative mRNA levels for B12 and B13. In a 10ul reaction, probes were used at 0.25nmol and primers for each probe were used at 0.5nmol per reaction (S2 Table). The single 10ul reaction also included about 40ng cDNA and 10ul of the 2X PrimeTime Gene Expression Master Mix (Integrated DNA Technologies). Each sample was completed in duplicate with three experimental replicates. The WT virus sample was used to generate a standard curved to determine amplification efficiency of the probe/primer sets and this number was factored into the cDNA fold values.

### Plasmid/siRNA/mRNA transfections

For plasmid transfection, CV1 cells in a 35mm well were transfected with 5μl lipofectamine2000 (Invitrogen) for 5μg pJS4-HA-B12wt or pJS4-HA-B12ΔA690 plasmid DNA following the manufacturer’s incubation suggestions. Cells were then infected with WT virus 6h post transfection for expression of HA-B12wt and HA-B12ΔA690 from the pJS4 vector under a late vaccinia virus promoter. For the transient depletion of B12 or B13 mRNA, CV1 cells in a 35mm well were transfected with a mixture of 5μl Lipofectamine RNAiMAX (Invitrogen) and 100nM siRNA (S2 Table) targeting the scramble control, B12, or B13 mRNA sequences. Transfected cells were incubated 24h at 37°C before infection of cells for downstream experiments. For mRNA transfections for immunofluorescence assays, in vitro synthesis of HA-GFP or HA-B12 mRNA was conducted following mMessage mMachine T7 Ultra manufacturer’s recommendations (Invitrogen) with linearized template pcDNA3.1-HA-GFP or pcDNA3.1-HA-B12 (S4B Fig). HA-GFP was cloned into the pcDNA3.1 vector using primers containing NheI or XhoI restriction sites (S2 Table). CV1 or CV1-B1myc cells were transfected with 1.5μl Lipofectamine MessengerMax (Invitrogen) and 1μg mRNA per well of a 12-well plate following the manufacturer’s protocol. The mRNA transfected cells were fixed or permeabilized the next day for immunofluorescence and prepermeabilization assays.

### Immunoblot assay

Protein expression was evaluated by harvesting cells and resuspending cells at 1 X 10^4^ or 5 X 10^3^ cells/μl in a 2X SDS protein sample buffer supplemented with 50 units/ml Pierce universal nuclease for cell lysis (Thermo Scientific), trypsin serine protease inhibitor (phenylmethylsulfonyl fluoride), protease inhibitor cocktail (Rocke), and phosphatase inhibitor cocktail (Roche). For detection of tubulin, HA epitope tagged proteins, lamin A/C and GAPDH, cells were resolved on a 12% SDS-PAGE gel. Total BAF and phosphorylated BAF protein was detected by resolving cells on an 18% SDS-PAGE gel. Transfer of the proteins to a polyvinylidene difluoride (PVDF) membrane were carried out overnight. Membranes were blocked in 5% milk made in 1X Tris buffer/NaCl/0.05% tween (1X TBST). Primary and secondary antibodies (S3 Table) added to 1% milk in 1XTBST were incubated with the membrane. Supersignal WestPico chemiluminescent reagents (Thermo Scientific) were incubated with the membranes. The Bio-Rad ImageLab software was used to quantify chemiluminescence signal. Images were made from film or chemidoc images. Relative B12 protein levels were averaged from 5 independent experiments. Raw values were quantified for HA-B12wt and HA-B12ΔA690 protein abundance using the volume tool in ImageLab software for chemidoc images. Control protein levels were used to normalize HA-B12wt or HA-B12ΔA690 raw values and to determine the relative B12 protein levels as graphed (Fig 3B). Relative phospho-BAF protein levels were quantified by dividing raw values for phosphorylated BAF from ImageLab volume tool by total BAF raw values for each experiment (S5A–S5C Fig) and averaged for the three experiments (S5D Fig).

### Immunofluorescence assay

Cells were fixed with 4% paraformaldehyde (Alfa Aesar) in 1X PBS for 15m and permeabilized with 0.2% Triton X-100 (Sigma) in 1X PBS for 10m. Primary antibodies were incubated with cells for 2h at room temperature (RT) following dilutions in 1X PBS (S3 Table). Secondary antibodies with conjugated fluorophore (S3 Table) were incubated with cells for 1h at RT in the dark. DAPI nuclear stain was added to cells at 1:1000 dilution in 1X PBS and incubated with cells for 30m at RT in the dark. Images were taken using an EVOS FL Auto Cell Imaging System (Invitrogen) with dual cameras and selected excitation/emission filters GFP (Fluor 488), TxRed (Fluor 594) and DAPI. ImageJ software was used for minor image editing.

### Prepermeabilization assay

CV1 cells were transfected with HA-GFP, HA-B12 or no mRNA following transfection protocol in section ‘Plasmid/siRNA/mRNA transfections’. 24h post transfection with mRNA cells were fixed with 4% paraformaldehyde (Alfa Aesar) in 1X PBS for control ‘Fix/Permeabilize Cells’ condition or first permeabilized with 0.1% Triton X-100 (Sigma) in 1X PBS for 30s, then fixed for ‘Permeabilize/Fix/Permeabilize Cells’ condition [46, 47]. The following steps were carried out identical to those stated in ‘Immunofluorescence assay’.

### Cellular fractionation assay

CV1 control cells or cells stably expressing HA-B12 were fractionated into soluble cytoplasmic (Cyto.), membrane (Memb.), nuclear (Nuc.), chromatin-bound (Chrom.) and cytoskeletal (Cytoskel.) fractions using the Subcellular Protein Fractionation Kit for Cultured Cells (Thermo Scientific #78840) following the manufacturer’s instructions with the addition of phosphatase inhibitors. Lamin A/C was used as a nuclear protein control that has soluble fractions and fractions bound to the chromatin and cytoskeleton. GAPDH and BAF are cytosolic and membrane associated protein controls. Additionally, the BAF control protein has a nuclear fraction that is chromatin-bound.

### Statistics

The error bars for each graph represent the standard deviation for values from the mean value. The *P* values were calculated using Prism multiple student *t* test or Excel student *t* test. Three or more experimental replicates were completed for each data figure except when stated otherwise.

## Supporting information

S1 Table Sequencing and mutation data from adapted ΔB1 viruses. (PDF)

S2 Table Probes, primers and siRNAs. (PDF)

S3 Table Antibodies and dilutions. (PDF)

S1 Fig Characterization of ΔB1 viruses serially passaged on CV1 cells. (A) Fold DNA abundance was quantified using qPCR and primers designed to vaccinia *HA* or *B1* genes for total viral DNA or B1 specific DNA. DNA was isolated from CV1 cells infected with WT, ΔB1, ΔB1-A1 passages 1–7, ΔB1-A2 passages 1–7, or ΔB1-A3 passages 1–7 viruses at a MOI of 3 and harvested 24h post infection. (B) Plaque assay of CV1 control or B1myc expressing cells infected with WT, ΔB1 and ΔB1-A1 virus from passages 1–7 at 200 PFU/well. Cells were fixed 72h post infection. (C) Experimental evolution depiction with genome reference identification numbers. There were no single nucleotide polymorphisms (SNPs) in >5% of the nucleotide read counts for the coding regions of vaccinia WR reference compared to WiebeLab virus genome, and WiebeLab compared to ΔB1 virus genome.
(TIF)

S2 Fig The ΔB1mutB12 viruses have a rescued phenotype in multiple cell lines. (A) Infections with WT (black), ΔB1 (red), ΔB1mutB12-A1 (light green), ΔB1mutB12-A3 (dark green) at a MOI of 3 were harvested 24h post infection for qPCR of relative DNA accumulation in HeLa, (B) A549, and (C) L929 cells or (D) for titration on CV1-B1myc cells for viral yield from infections of HeLa, (E) A549, or (F) L929 cells.
(TIF)

S3 Fig Depletion of B12 or B13 mRNA impact on neighboring gene expression and virus plaque formation. (A) Depiction of *B12R* and *B13R* general regions targeted by siRNA for mRNA depletion and probe/primer set binding of cDNA to quantify relative early gene expression using qPCR. (B) CV1 cells were transfected with siRNA for 24h then infected with WT (black), ΔB1 (red), or ΔB1mutB12-A3 (green) at a MOI of 3 and harvested 4h post infection for mRNA isolation. The cDNA generated from harvested mRNA samples was used with probe/primer sets to quantify early gene expression for *B12R* and (C) *B13R* using probe/primers B13R.1 set or (D) B13R.2 set. (E) Plaque assay of CV1 cells transfected with siRNA for 24h were infected with WT, ΔB1 or ΔB1-A3 virus at 200 PFU/well and fixed 72h post infection.
(TIF)

S4 Fig Sequences for vaccinia B12R codon optimized for expression in mammalian cells. (A) A vaccinia *B12R* gene codon optimized for expression in mammalian cells was generated by GeneArt and (B) GenScript.
(TIF)

S5 Fig ΔB1mutB12 virus infection enhances BAF phosphorylation as compared to ΔB1 virus infection. (A) Lysates from CV1 cells uninfected (grey) or infected with WT (black), ΔB1 (red), ΔB1mutB12-A1 (light green), or ΔB1mutB12-A3 (dark green) were subjected to immunoblot analysis of total BAF protein and phosphorylated BAF. Protein levels were determined by chemiluminescence quantification using ImageLab on chemidoc images and raw values were used to calculate phospho-BAF over total BAF levels for biological replicate experiment 1, (B) experiment 2, and (C) experiment 3. (D) The phospho-BAF levels relative to total BAF levels were averaged for all three experiments.
(TIF)
