# Supplementary material for: A poxvirus pseudokinase represses viral DNA replication via a pathway antagonized by its paralog kinase
Source: PLoS Pathog. 2019 Feb 15;15(2):e1007608. doi: 10.1371/journal.ppat.1007608 (PMC6395007; doi:10.1371/journal.ppat.1007608)
Supplement: S1 Table — (PDF) [file ppat.1007608.s001.pdf]

Table S1. Sequnencing data from adapted ΔB1 viruses

Whole Genome Illumina Sequencing Data (Mutations >5%)

Vaccinia Virus	Mutation Type	WT WR Ref.[c]	B12R[d]	Read Depth	Mutation	Mutation %[e]	A.A. Sequence Change[f]	Notes
ΔB1-A1[a]	SNP	173172 bp	644 bp	1077	G → A	18.15%	G → D at 215 a.a.	Mutation not detected in ΔB1-A3 virus (read depth: 1146)
ΔB1-A1[a]	Indel	172854 bp	326 bp	1667	+ C	2.52%	Premature STOP at 113 a.a.	Indel occurs in a run of four cytosines
					- C	2.46%	Premature STOP at 122 a.a.	Indel occurs in a run of four cytosines
ΔB1-A3[b]				1794	+ C	1.90%	Premature STOP at 113 a.a.	Indel occurs in a run of four cytosines (under 5% threshold)
					- C	2.29%	Premature STOP at 122 a.a.	Indel occurs in a run of four cytosines (under 5% threshold)
ΔB1-A1[a]	Indel	173217 bp	689 bp	973	+ A	36.07%	Premature STOP at 234 a.a.	Indel occurs in a run of eight adenines
					- A	11.92%	Premature STOP at 237 a.a.	Indel occurs in a run of eight adenines
ΔB1-A3[b]				1104	+ A	50.09%	Premature STOP at 234 a.a.	Indel occurs in a run of eight adenines
					- A	15.76%	Premature STOP at 237 a.a.	Indel occurs in a run of eight adenines

Targeted *B12R* Sanger Sequencing Data

Virus	Isolate	Mutation Type	WT WR Ref.[c]	B12R[d]	Mutation	A.A. Sequence Change[f]	Notes
ΔB1-A1	1	Indel	173217 bp	689 bp	+ A	Premature STOP at 234 a.a.	Indel occurs in a run of eight adenines
ΔB1-A1	2				- A	Premature STOP at 237 a.a.	Indel occurs in a run of eight adenines
ΔB1-A2	1	Indel	172942 bp	414 bp	+ A[g]	Premature STOP at 147 a.a.	Indel occurs in a run of five adenines
ΔB1-A2	2	Indel	173217 bp	689 bp	+ A	Premature STOP at 234 a.a.	Indel occurs in a run of eight adenines
ΔB1-A2	3				+ A		Indel occurs in a run of eight adenines
ΔB1-A2	4				+ A		Indel occurs in a run of eight adenines
ΔB1-A2	5				- A	Premature STOP at 237 a.a.	Indel occurs in a run of eight adenines
ΔB1-A3	1	Indel	172854 bp	326 bp	+ C[g]	Premature STOP at 113 a.a.	Indel occurs in a run of four cytosines
ΔB1-A3	2	Indel	173217 bp	689 bp	+ A	Premature STOP at 234 a.a.	Indel occurs in a run of eight adenines
ΔB1-A3	3				- A	Premature STOP at 237 a.a.	Indel occurs in a run of eight adenines

[a] ΔB1-A1 Genome (Genbank SAMN10039698)

[b] ΔB1-A3 Genome (Genbank SAMN10039767)

[c] Site of nucleotide mutation within the WT WR Reference Genome (Genbank AY243312.1)

[d] Site of nucleotide mutation within the *B12R* gene relative to the ATG start site (NC_006998.1)

[e] Mutation percentage is calculated by dividing nucleotide reads containing a mutation by the read depth at the specific nuclotide site.

[f] Premature STOP results in an amino acid sequnce less than the full length 283 a.a. B12 protein.

[g] Isolate lacks insertion/deletion mutation at 689 bp site within the *B12R* gene.
