# Supplementary material for: A poxvirus pseudokinase represses viral DNA replication via a pathway antagonized by its paralog kinase
Source: PLoS Pathog. 2019 Feb 15;15(2):e1007608. doi: 10.1371/journal.ppat.1007608 (PMC6395007; doi:10.1371/journal.ppat.1007608)
Supplement: S2 Table — (PDF) [file ppat.1007608.s002.pdf]

**Table S2. Probes, primers and siRNAs**

| Name | Sequence |
|---|---|
| B11R F Primer | 5’-TGCTTACTACTAACATGGATACAGA-3’ |
| B13R R Pimer | 5’-TCAATACTGACGAGATTGAC-3’ |
| B12R F Primer (Sanger Seq) | 5’-CCAGATCTGTATGGAATTGGAGAAACCG-3’ |
| B12R R Primer (Sanger Seq) | 5’-CCTCGGTTCTATTTTTCCATGGG-3’ |
| Total VACV DNA (HA) F Primer | 5’-CATCATCTGGAATTGTCACTACTAAA-3’ |
| Total VACV DNA (HA) R Primer | 5’-ACGGCCGACAATTATAATTAATGC-3’ |
| B1R F Primer | 5’-GTGCAAGGCATTTGGTCTATAC-3’ |
| B1R R Primer | 5’-CAACATCACCGACCTTTTTGG-3’ |
| B12R Probe | 5’-/56-FAM/TTGGAGCAA/ZEN/CAGTTTCAA-3’ |
| B12R F Primer (qPCR) | 5’-ACTCACATATAGATTACAACGAGGAC-3’ |
| B12R R Primer (qPCR) | 5’-ACCGAACCATTCTATCATGCA-3’ |
| B13R.1 Probe | 5’-/56-FAM/AGCTGTTCA/ZEN/GCAGTGGAT-3’ |
| B13R.1 F Primer (qPCR) | 5’-CAGCGTCAATCTCGTCAGTAT-3’ |
| B13R.1 R Primer (qPCR) | 5’-CCTTATCCATGTTCTCCTCCTTT-3’ |
| B13R.2 Probe | 5’-/56-FAM/ACAGAGGTG/ZEN/TTCGGTTCA-3’ |
| B13R.2 F Primer (qPCR) | 5’-GGCTCGTATAATCTGGTGGATAC-3’ |
| B13R.2 R Primer (qPCR) | 5’-CGTCGACACTCACATCTGAATTA-3’ |
| BamHI-Kozak-HA-B12 F Primer | 5'-GAGAGAGGATCCGCCACCATGTATCCCTACGACG-3' |
| BamHI-Kozak-B12 F Primer | 5'-GAGAGAGGATCCGCCACCATGGAAAGCTTCAAGTACTG-3' |
| B12-BamHI R Primer | 5'-GAGAGAGGATCCTTAGTCCTGGATGAACAGCTTCCGC-3' |
| XhoI-Kozak-HA-B12R F Primer | 5'-ATTATCTCGAGGCCACCATGTACCCTTATGATGTGCCAGACTATGCTATGGAATCCTTCAAGTATTGTTTTGATAACG-3' |
| B12R-NheI R Primer | 5'-GACTAGCTAGCTCAATCTTGTATAAACAGTTTACGTAGTC-3' |
| TK-locus L Primer | 5'-GGGACTATGGACGCATGATAAG-3' |
| TK-locus R Primer | 5'-ACACTTTCTACACACCGATTGA-3' |
| TK L Primer | 5'-ATACGGAACGGGACTATGGA-3' |
| B12R F Primer | 5'-ACAGTTTCAAGACGAGGAGATTTA-3' |
| NheI-Kozak-HA-eGFP F Primer | 5'-ATTATGCTAGCGCCACCATGTACCCTTATGATGTGCCTGATTATGCAATGGTGAGCAAGGGCGAGG-3' |
| eGFP-XhoI R Primer | 5'-TCACACTCGAGTTACTTGTACAGCTCGTCC-3' |
| siCtrl (Scramble) | 5’-CAGUCGCGUUUGCGACUGGUU-3’ |
| siB12 (siB12-1) | 5’-GGUAUAAAGUAUUUGGCUAUU-3’ |
| siB12-2 | 5’-CAUGAUAACUUCAGGAAAUUU-3’ |
| siB12-3 | 5’-GGAUAUUGCAUGAUAGAAUUU-3’ |
| siB12-4 | 5’-UGAUAACGAUGGCAAGAAAUU-3’ |
| siB13-1 | 5’-AGACAAGAUUGAUGGAUUAUU-3’ |
| siB13-2 | 5’-GGAUAAGGUUAGCGCUCAAUU-3’ |
