# Supplementary material for: A poxvirus pseudokinase represses viral DNA replication via a pathway antagonized by its paralog kinase
Source: PLoS Pathog. 2019 Feb 15;15(2):e1007608. doi: 10.1371/journal.ppat.1007608 (PMC6395007; doi:10.1371/journal.ppat.1007608)
Supplement: S3 Table — (PDF) [file ppat.1007608.s003.pdf]

Table S3. Antibodies and dilutions

Antibody	Company	Clone	Assay/Dilution
αBAF (total) (rabbit)	custom		IB(1:3,000)
αGAPDH (mouse)	Santa Cruz Biotechnology		IB(1:200)
αHA.11 (mouse)	BioLegend	16B12	IFA(1:400)/ WB(1:1,000)
αI3 (rabbit)	custom		IFA(1:300)
αLaminA/C (mouse)	Cell Signaling		IB(1:2,000)
αmyc (mouse)	Cell Signaling	9B11	IFA(1:100)
αphospho-BAF (rabbit)	custom		IB(1:1,000)
αtubulin (mouse)	Sigma Aldrich	T7816	IB(1:10,000)
Goat αmouse	BioRad		IB(1:20,000)
Goat αrabbit	BioRad		IB(1:20,000)
Fluor 594 OR 488 conjugated goat αmouse	Life Technologies		IFA(1:400)
Fluor 488 conjugated goat αrabbit	Life Technologies		IFA(1:400)
