# Supplementary figures and images for: A poxvirus pseudokinase represses viral DNA replication via a pathway antagonized by its paralog kinase
Source: PLoS Pathog. 2019 Feb 15;15(2):e1007608. doi: 10.1371/journal.ppat.1007608 (PMC6395007; doi:10.1371/journal.ppat.1007608)

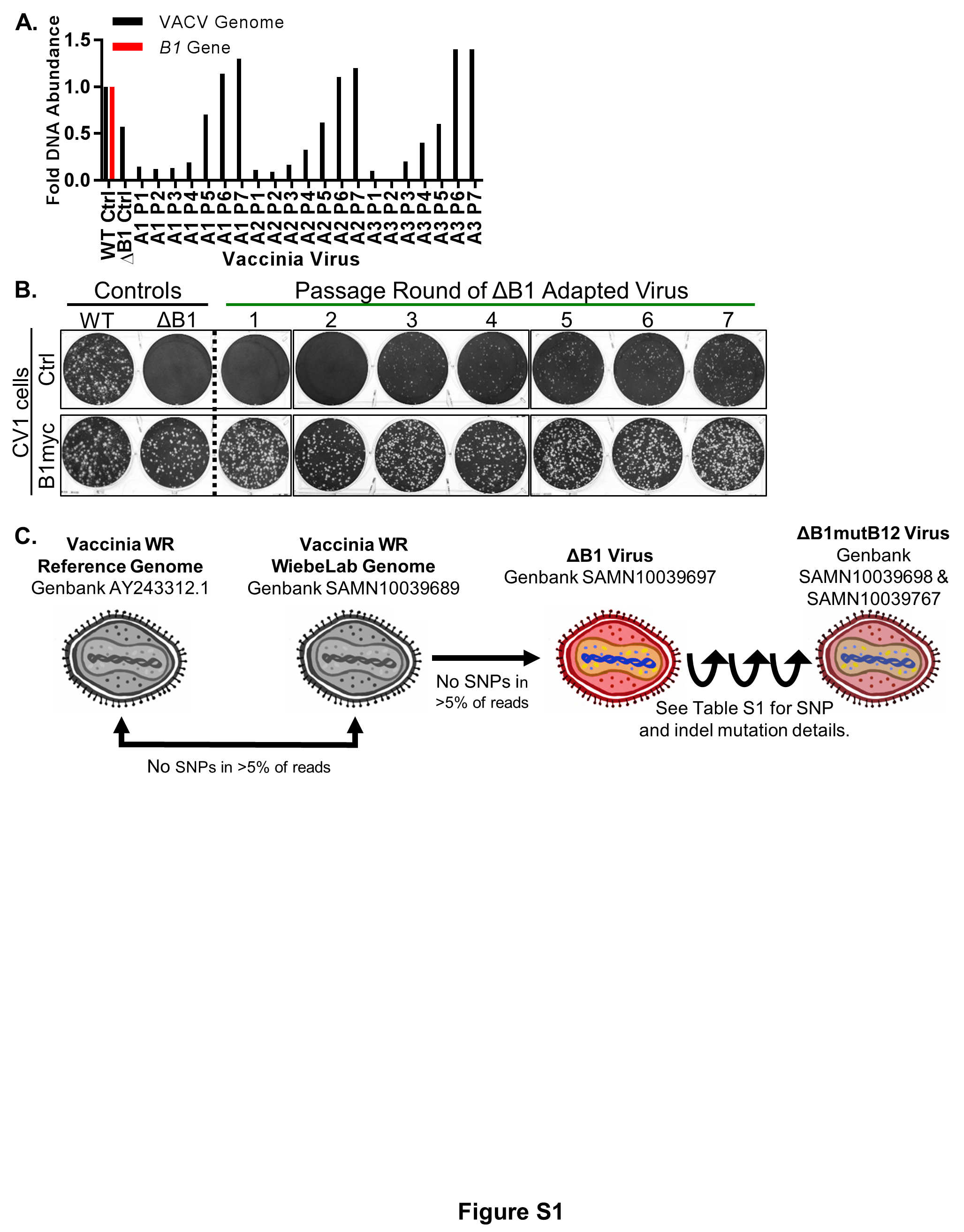

Supplement: S1 Fig — (A) Fold DNA abundance was quantified using qPCR and primers designed to vaccinia HA or B1 genes for total viral DNA or B1 specific DNA. DNA was isolated from CV1 cells infected with WT, ΔB1, ΔB1-A1 passages 1–7, ΔB1-A2 passages 1–7, or ΔB1-A3 passages 1–7 viruses at a MOI of 3 and harvested 24h post infection. (B) Plaque assay of CV1 control or B1myc expressing cells infected with WT, ΔB1 and ΔB1-A1 virus from passages 1–7 at 200 PFU/well. Cells were fixed 72h post infection. (C) Experimental evolution depiction with genome reference identification numbers. There were no single nucleotide polymorphisms (SNPs) in >5% of the nucleotide read counts for the coding regions of vaccinia WR reference compared to WiebeLab virus genome, and WiebeLab compared to ΔB1 virus genome. (TIF) [file ppat.1007608.s004.tif]

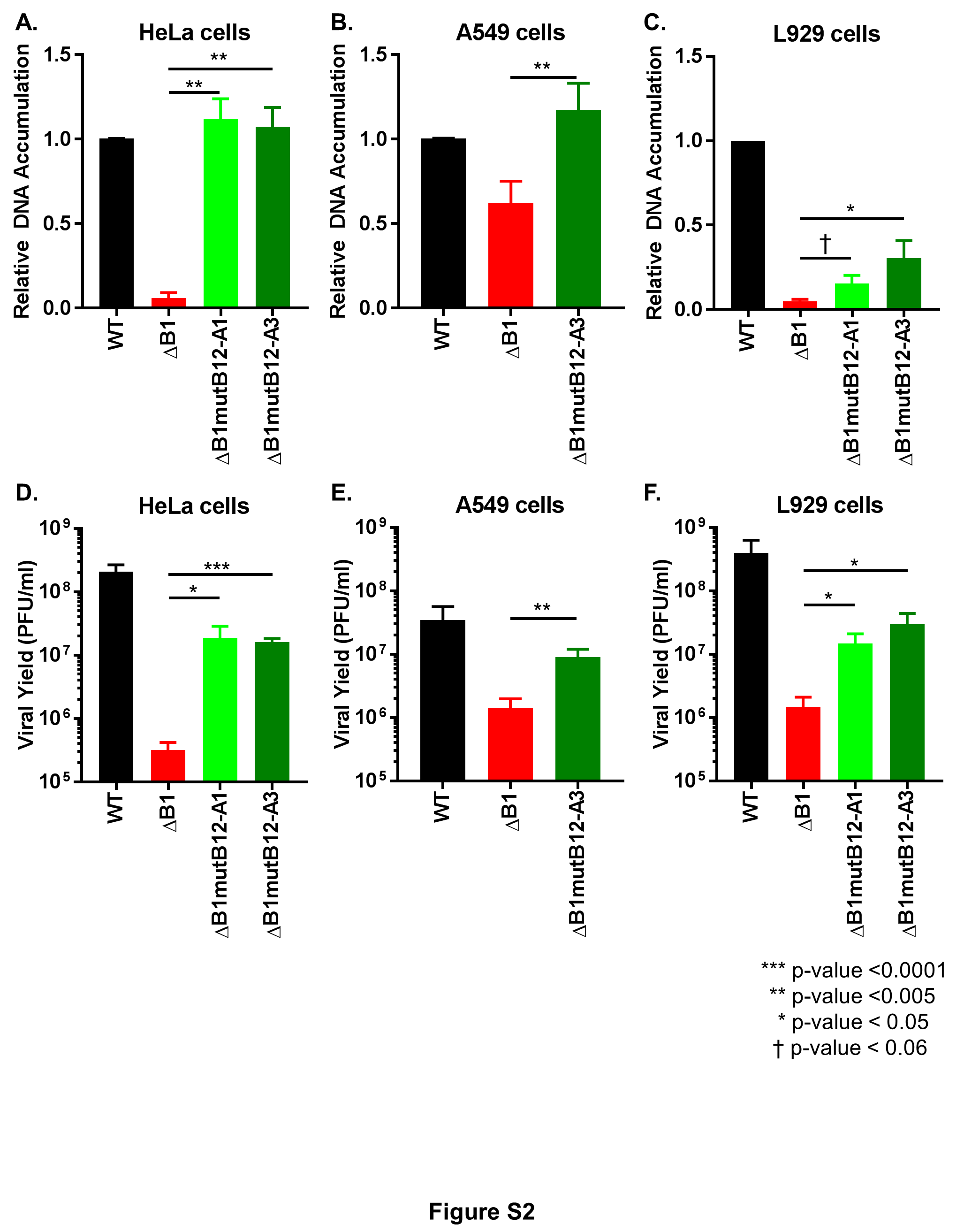

Supplement: S2 Fig — (A) Infections with WT (black), ΔB1 (red), ΔB1mutB12-A1 (light green), ΔB1mutB12-A3 (dark green) at a MOI of 3 were harvested 24h post infection for qPCR of relative DNA accumulation in HeLa, (B) A549, and (C) L929 cells or (D) for titration on CV1-B1myc cells for viral yield from infections of HeLa, (E) A549, or (F) L929 cells. (TIF) [file ppat.1007608.s005.tif]

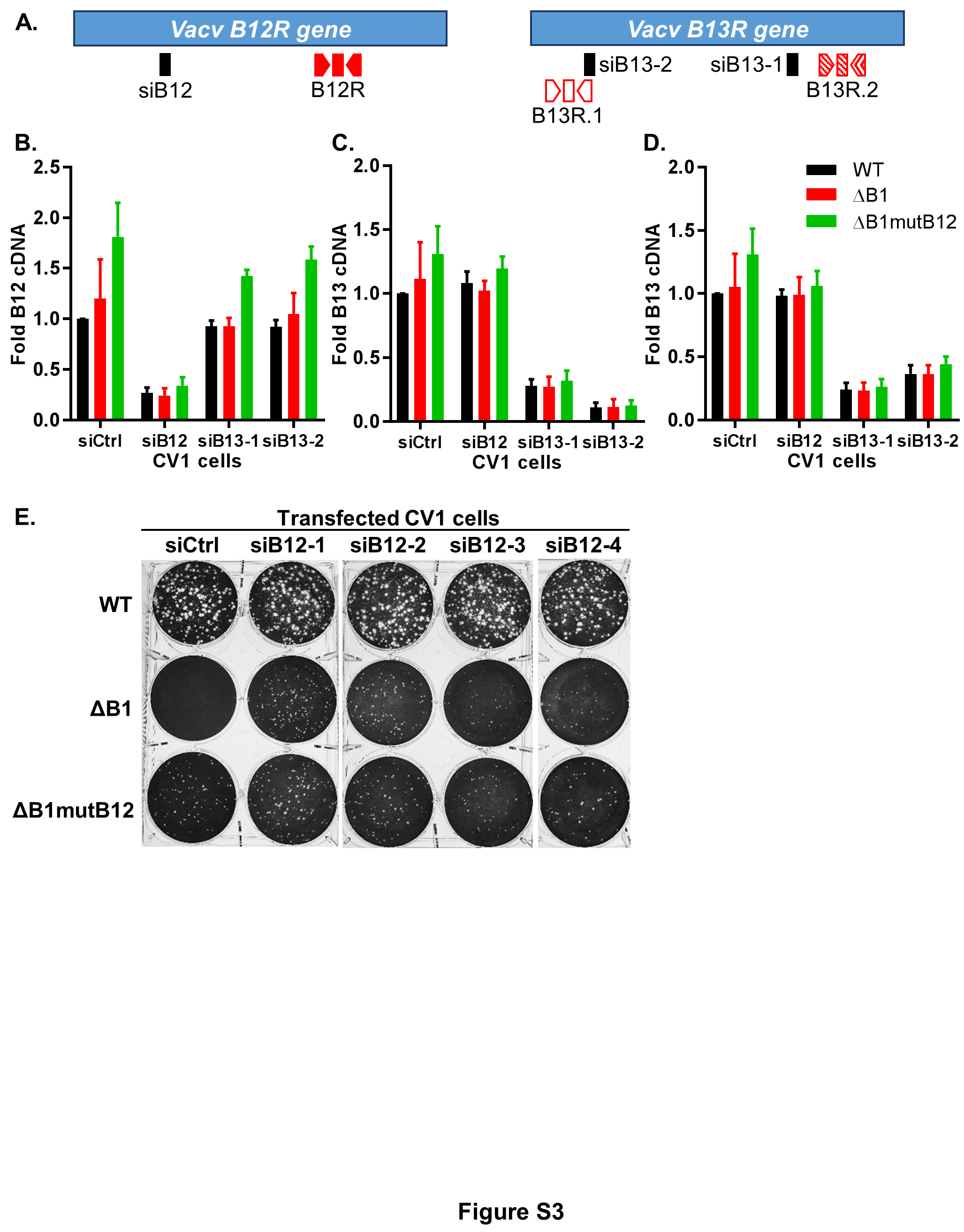

Supplement: S3 Fig — (A) Depiction of B12R and B13R general regions targeted by siRNA for mRNA depletion and probe/primer set binding of cDNA to quantify relative early gene expression using qPCR. (B) CV1 cells were transfected with siRNA for 24h then infected with WT (black), ΔB1 (red), or ΔB1mutB12-A3 (green) at a MOI of 3 and harvested 4h post infection for mRNA isolation. The cDNA generated from harvested mRNA samples was used with probe/primer sets to quantify early gene expression for B12R and (C) B13R using probe/primers B13R.1 set or (D) B13R.2 set. (E) Plaque assay of CV1 cells transfected with siRNA for 24h were infected with WT, ΔB1 or ΔB1-A3 virus at 200 PFU/well and fixed 72h post infection. (TIF) [file ppat.1007608.s006.tif]

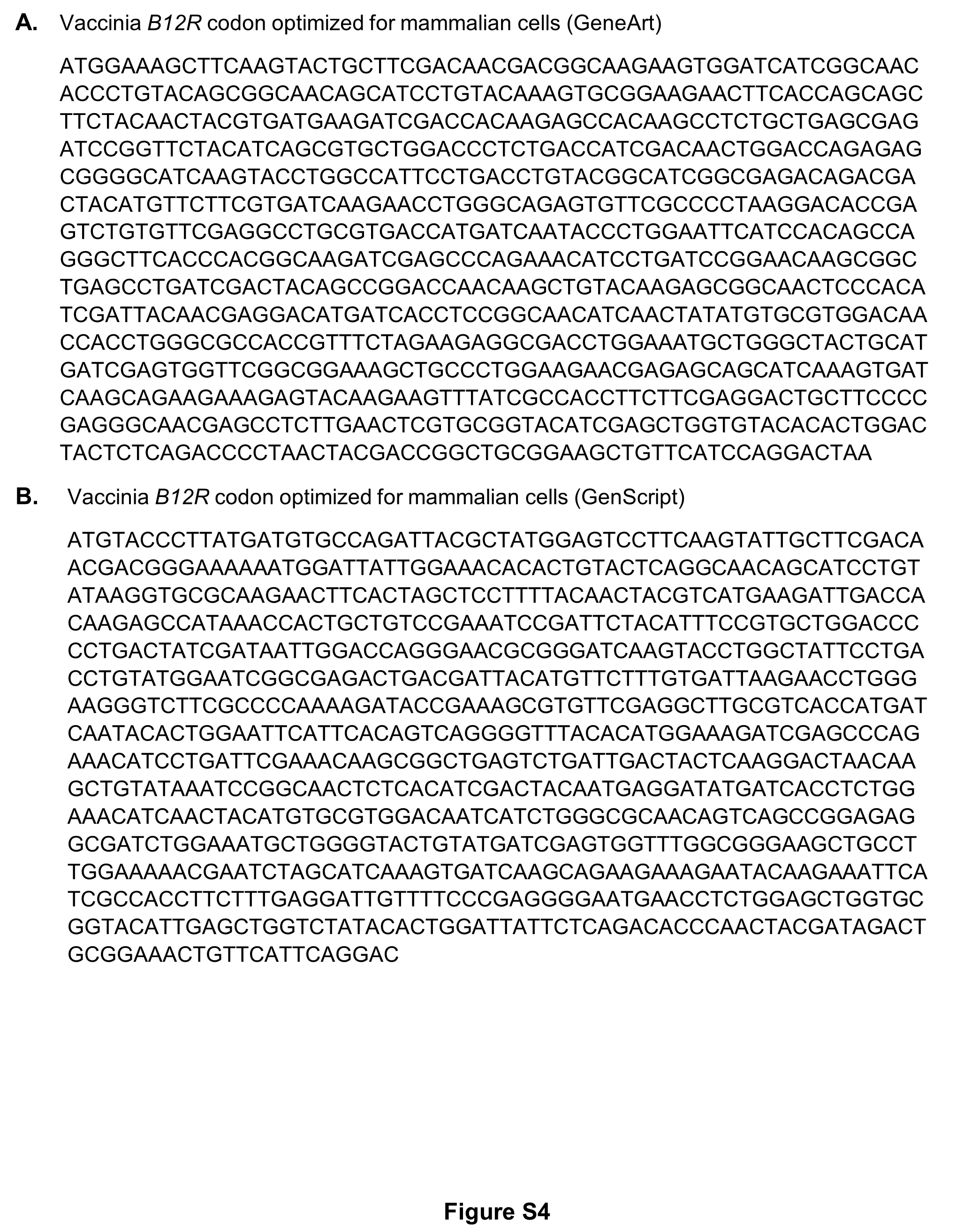

Supplement: S4 Fig — (A) A vaccinia B12R gene codon optimized for expression in mammalian cells was generated by GeneArt and (B) GenScript. (TIF) [file ppat.1007608.s007.tif]

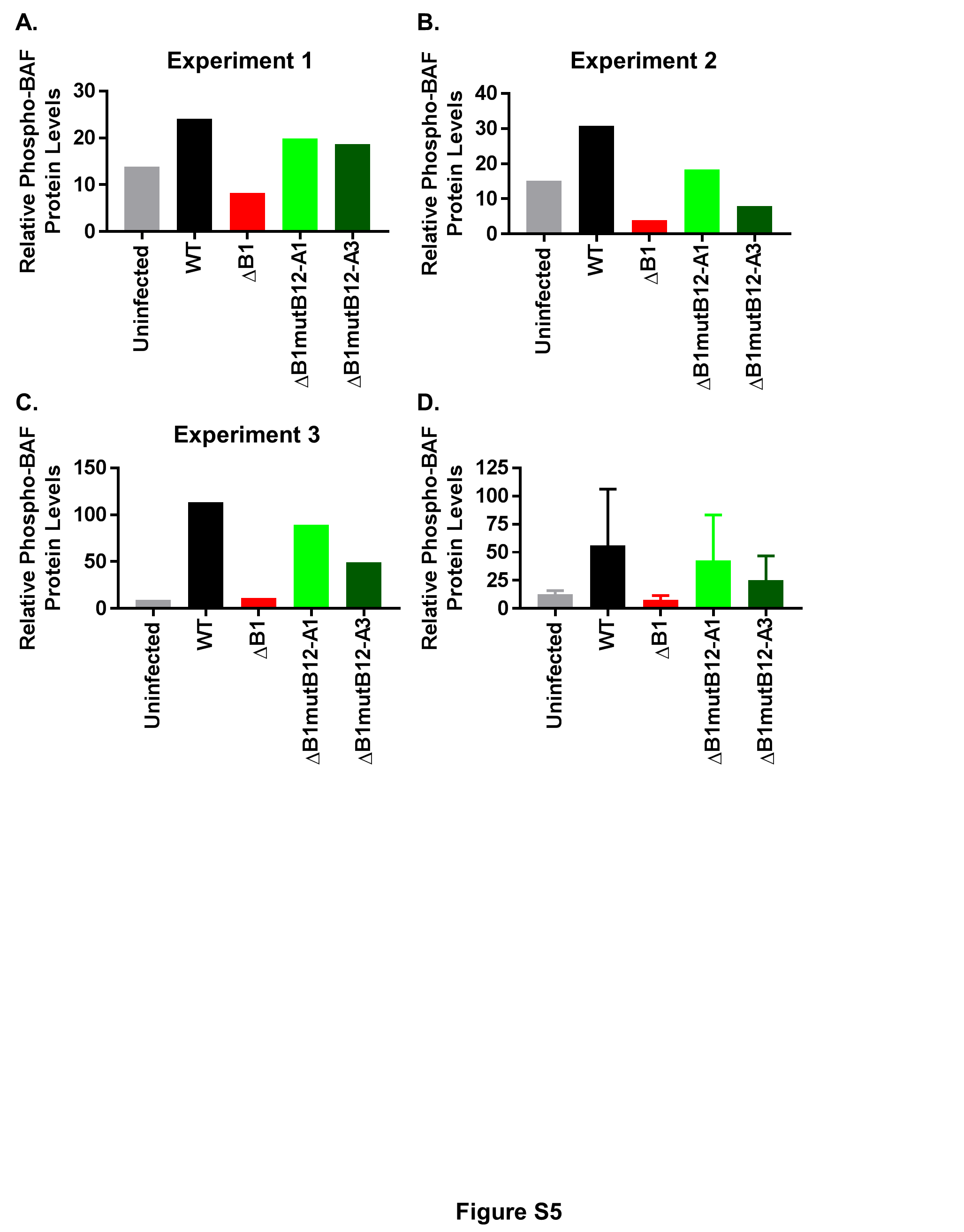

Supplement: S5 Fig — (A) Lysates from CV1 cells uninfected (grey) or infected with WT (black), ΔB1 (red), ΔB1mutB12-A1 (light green), or ΔB1mutB12-A3 (dark green) were subjected to immunoblot analysis of total BAF protein and phosphorylated BAF. Protein levels were determined by chemiluminescence quantification using ImageLab on chemidoc images and raw values were used to calculate phospho-BAF over total BAF levels for biological replicate experiment 1, (B) experiment 2, and (C) experiment 3. (D) The phospho-BAF levels relative to total BAF levels were averaged for all three experiments. (TIF) [file ppat.1007608.s008.tif]
